# Identification of polymorphisms in 12q24.1, *ACAD10*, and *BRAP* as novel genetic determinants of blood pressure in Japanese by exome-wide association studies

**DOI:** 10.18632/oncotarget.17474

**Published:** 2017-04-27

**Authors:** Yoshiji Yamada, Jun Sakuma, Ichiro Takeuchi, Yoshiki Yasukochi, Kimihiko Kato, Mitsutoshi Oguri, Tetsuo Fujimaki, Hideki Horibe, Masaaki Muramatsu, Motoji Sawabe, Yoshinori Fujiwara, Yu Taniguchi, Shuichi Obuchi, Hisashi Kawai, Shoji Shinkai, Seijiro Mori, Tomio Arai, Masashi Tanaka

**Affiliations:** ^1^ Department of Human Functional Genomics, Advanced Science Research Promotion Center, Mie University, Tsu, Japan; ^2^ CREST, Japan Science and Technology Agency, Kawaguchi, Japan; ^3^ Computer Science Department, College of Information Science, University of Tsukuba, Tsukuba, Japan; ^4^ RIKEN Center for Advanced Intelligence Project, Tokyo, Japan; ^5^ Department of Computer Science, Nagoya Institute of Technology, Nagoya, Japan; ^6^ Department of Internal Medicine, Meitoh Hospital, Nagoya, Japan; ^7^ Department of Cardiology, Kasugai Municipal Hospital, Kasugai, Japan; ^8^ Department of Cardiovascular Medicine, Inabe General Hospital, Inabe, Japan; ^9^ Department of Cardiovascular Medicine, Gifu Prefectural Tajimi Hospital, Tajimi, Japan; ^10^ Department of Molecular Epidemiology, Medical Research Institute, Tokyo Medical and Dental University, Tokyo, Japan; ^11^ Section of Molecular Pathology, Graduate School of Health Care Sciences, Tokyo Medical and Dental University, Tokyo, Japan; ^12^ Research Team for Social Participation and Community Health, Tokyo Metropolitan Institute of Gerontology, Tokyo, Japan; ^13^ Research Team for Promoting Support System for Home Care, Tokyo Metropolitan Institute of Gerontology, Tokyo, Japan; ^14^ Research Team for Social Participation and Health Promotion, Tokyo Metropolitan Institute of Gerontology, Tokyo, Japan; ^15^ Center for Promotion of Clinical Investigation, Tokyo Metropolitan Geriatric Hospital, Tokyo, Japan; ^16^ Department of Pathology, Tokyo Metropolitan Geriatric Hospital, Tokyo, Japan; ^17^ Department of Clinical Laboratory, Tokyo Metropolitan Geriatric Hospital, Tokyo, Japan

**Keywords:** hypertension, blood pressure, genetics, exome-wide association study, polymorphism

## Abstract

We performed exome-wide association studies to identify genetic variants that influence systolic or diastolic blood pressure or confer susceptibility to hypertension in Japanese. The exome-wide association studies were performed with the use of Illumina HumanExome-12 DNA Analysis BeadChip or Infinium Exome-24 BeadChip arrays and with 14,678 subjects, including 8215 individuals with hypertension and 6463 controls. The relation of genotypes of 41,843 single nucleotide polymorphisms to systolic or diastolic blood pressure was examined by linear regression analysis. After Bonferroni's correction, 44 and eight polymorphisms were significantly (*P* < 1.19 × 10^−6^) associated with systolic or diastolic blood pressure, respectively, with six polymorphisms (rs12229654, rs671, rs11066015, rs2074356, rs3782886, rs11066280) being associated with both systolic and diastolic blood pressure. Examination of the relation of allele frequencies to hypertension with Fisher's exact test revealed that 100 of the 41,843 single nucleotide polymorphisms were significantly (*P* < 1.19 × 10^−6^) associated with hypertension. Subsequent multivariable logistic regression analysis with adjustment for age and sex showed that five polymorphisms (rs150854849, rs202069030, rs139012426, rs12229654, rs76974938) were significantly (*P* < 1.25 × 10^−4^) associated with hypertension. The polymorphism rs12229654 was thus associated with both systolic and diastolic blood pressure and with hypertension. Six polymorphisms (rs12229654 at 12q24.1, rs671 of *ALDH2*, rs11066015 of *ACAD10*, rs2074356 and rs11066280 of *HECTD4*, and rs3782886 of *BRAP*) were found to be associated with both systolic and diastolic blood pressure, with those at 12q24.1 or in *ACAD10* or *BRAP* being novel determinants of blood pressure in Japanese.

## INTRODUCTION

Hypertension is a major risk factor for coronary artery disease and stroke [1], with a heritability estimated to be ~30% [2]. Genome-wide association studies (GWASs) have implicated various loci and genes in predisposition to hypertension in populations of European [3–6] or African [7] ancestry as well as in Chinese [8] and Japanese [9] individuals. A recent trans-ancestry GWAS in individuals of European and East or South Asian ancestry identified 12 loci associated with blood pressure (BP) [10]. Most of the genetic variants identified in these studies have a minor allele frequency (MAF) of ≥5% and a small individual effect size. Given that these common variants explain only a fraction of the heritability of hypertension, low-frequency (0.5% ≤ MAF < 5%) or rare (MAF < 0.5%) variants with larger effect sizes likely contribute to the genetic architecture of this condition [11].

We have now performed exome-wide association studies (EWASs) with the use of exome array–based genotyping methods to identify single nucleotide polymorphisms (SNPs)—in particular, low-frequency or rare coding variants with moderate to large effect sizes—that confer susceptibility to elevated BP or hypertension in Japanese. We applied Illumina human exome arrays that provide coverage of functional SNPs in entire exons including low-frequency and rare variants.

## RESULTS

### EWASs for systolic and diastolic BP

We examined the relation of genotypes for 41,843 SNPs that passed quality control to systolic or diastolic BP by linear regression analysis. Manhattan plots of the EWASs for systolic and diastolic BP are shown in Supplementary Figure 1. After Bonferroni's correction, 44 or eight SNPs were significantly (*P* < 1.19 × 10^−6^) associated with systolic (Table 1) or diastolic (Table 2) BP, respectively. Among these SNPs, six polymorphisms (rs12229654 at 12q24.1, rs671 of *ALDH2*, rs11066015 of *ACAD10*, rs2074356 and rs11066280 of *HECTD4*, rs3782886 of *BRAP*) were significantly associated with both systolic and diastolic BP.

**Table 1:** The 44 single nucleotide polymorphisms (SNPs) significantly (*P* < 1.19 × 10^−^^6^) associated with systolic blood pressure by the exome-wide association study

Gene	dbSNP	Nucleotide (amino acid) substitution^a^	Chromosome: position	MAF (%)	*P* (genotype)
*MUC17*	rs78010183	A/T (T1305S)	7: 101035329	1.8	3.86 × 10^−32^
*OR4F6*	rs141569282	G/A (A117T)	15: 101806068	1.7	1.10 × 10^−27^
*COL6A5*	rs200982668	G/A (E2501K)	3: 130470894	1.3	2.45 × 10^−22^
*MARCH1*	rs61734696	G/T (Q137K)	4: 164197303	1.2	3.48 × 10^−22^
*PLCB2*	rs200787930	C/T (E1095K)	15: 40289298	1.2	6.54 × 10^−22^
*MOB3C*	rs139537100	C/T (R24Q)	1: 46615006	1.2	2.54 × 10^−21^
*VPS33B*	rs199921354	C/T (R80Q)	15: 91013841	1.2	4.06 × 10^−21^
*CXCL8*	rs188378669	G/T (E31*)	4: 73741568	1.2	1.40 × 10^−20^
*COL6A3*	rs146092501	C/T (E1386K)	2: 237371861	1.2	2.45 × 10^−20^
*ZNF77*	rs146879198	G/A (R340*)	19: 2934109	1.2	2.70 × 10^−20^
*TMOD4*	rs115287176	G/A (R277W)	1: 151170961	1.2	5.08 × 10^−20^
*ADGRL3*	rs192210727	G/T (R580I)	4: 61909615	1.3	1.20 × 10^−19^
*PRAMEF12*	rs199576535	G/A (V341I)	1: 12777168	1.0	5.70 × 10^−16^
*PTCH2*	rs147284320	C/T (V503I)	1: 44828589	2.0	4.36 × 10^−14^
*IGSF9B*	rs201459911	G/A (A1115V)	11: 133920381	0.7	6.77 × 10^−14^
	rs12229654	T/G	12: 110976657	22.5	2.04 × 10^−11^
*ALDH2*	rs671	G/A (E504K)	12: 111803962	27.6	3.97 × 10^−11^
*ACAD10*	rs11066015	G/A	12: 111730205	27.5	8.38 × 10^−11^
*HECTD4*	rs2074356	C/T	12: 112207597	25.4	4.48 × 10^−10^
*BRAP*	rs3782886	A/G	12: 111672685	29.3	2.82 × 10^−9^
*HECTD4*	rs11066280	T/A	12: 112379979	29.0	3.38 × 10^−9^
*RNF213*	rs199976159	G/A (G222S)	17: 80288217	0.3	3.98 × 10^−9^
	rs2523638	G/A	6: 31376496	43.1	1.12 × 10^−8^
*AS3MT*	rs11191454	A/G	10: 102900247	26.8	2.39 × 10^−8^
	rs12182351	T/C	6: 32233930	29.8	2.42 × 10^−8^
*CNNM2*	rs12413409	G/A	10: 102959339	26.4	3.24 × 10^−8^
*NPFFR2*	rs144936999	G/C (A332P)	4: 72147237	0.2	4.16 × 10^−8^
	rs404890	G/T	6: 32231090	30.5	4.94 × 10^−8^
*CCHCR1*	rs130075	C/T (R102Q)	6: 31154725	13.2	5.07 × 10^−8^
*NT5C2*	rs11191580	T/C	10: 103146454	26.3	6.68 × 10^−8^
*HLA-B*	rs1058026	T/G	6: 31353908	33.4	9.03 × 10^−8^
*CNNM2*	rs11191548	T/C	10: 103086421	26.3	9.31 × 10^−8^
*C6orf15*	rs2270191	C/T (V5M)	6: 31112543	12.4	1.79 × 10^−7^
*CDSN*	rs117951780	C/T (S453N)	6: 31116257	12.3	2.45 × 10^−7^
*C6orf15*	rs2270190	T/C	6: 31112809	12.9	2.96 × 10^−7^
*CYP17A1*	rs17115100	G/T	10: 102831636	33.5	3.44 × 10^−7^
	rs72655343	C/A	11: 1104741	0.1	4.24 × 10^−7^
*PSORS1C1*	rs1063646	C/T (P133L)	6: 31139871	12.9	5.26 × 10^−7^
*CCHCR1*	rs9263739	C/T	6: 31143579	12.9	5.26 × 10^−7^
*CYP17A1*	rs1004467	T/C	10: 102834750	32.4	5.63 × 10^−7^
*CAT*	rs139421991	G/A (R320Q)	11: 34456720	0.3	6.74 × 10^−7^
*PSORS1C2*	rs7757012	T/C	6: 31140785	13.0	8.23 × 10^−7^
*RNF39*	rs142979264	C/T	6: 30075223	11.8	9.58 × 10^−7^
*CCDC63*	rs10849915	T/C	12: 110895818	23.6	1.18 × 10^−6^

The relation of genotypes of SNPs to systolic blood pressure was analyzed with a linear regression model. ^a^Major allele/minor allele. MAF, minor allele frequency.

**Table 2:** The eight single nucleotide polymorphisms (SNPs) significantly (*P* < 1.19 × 10^−^^6^) associated with diastolic blood pressure by the exome-wide association study

Gene	dbSNP	Nucleotide (amino acid) substitution^a^	Chromosome: position	MAF (%)	*P* (genotype)
*ALDH2*	rs671	G/A (E504K)	12: 111803962	27.6	2.18 × 10^−15^
*ACAD10*	rs11066015	G/A	12: 111730205	27.5	4.08 × 10^−15^
*BRAP*	rs3782886	A/G	12: 111672685	29.3	2.46 × 10^−14^
*HECTD4*	rs11066280	T/A	12: 112379979	29.0	8.38 × 10^−14^
*HECTD4*	rs2074356	C/T	12: 112207597	25.4	2.41 × 10^−13^
	rs12229654	T/G	12: 110976657	22.5	2.16 × 10^−12^
*ATXN2*	rs7969300	T/C (N248S)	12: 111555908	38.8	2.62 × 10^−8^
*NAA25*	rs12231744	C/T (R876K)	12: 112039251	35.1	6.76 × 10^−7^

The relation of genotypes of SNPs to diastolic blood pressure was analyzed with a linear regression model. ^a^Major allele/minor allele. MAF, minor allele frequency.

### EWAS for hypertension

We next performed an EWAS for hypertension. The characteristics of the subjects enrolled in the study are shown in Table 3. Age, the frequency of men, body mass index, and the prevalence of diabetes mellitus, dyslipidemia, chronic kidney disease, and hyperuricemia were greater, whereas the prevalence of smoking was lower, in subjects with hypertension than in controls. We examined the relation of allele frequencies of 41,843 SNPs to hypertension with Fisher's exact test. A Manhattan plot of the EWAS for hypertension is shown in Supplementary Figure 1. After Bonferroni's correction, 100 SNPs were significantly (*P* < 1.19 × 10^−6^) associated with hypertension (Supplementary Table 1). The genotype distributions of these SNPs were in Hardy-Weinberg equilibrium (*P* ≥ 0.001) among both subjects with hypertension and controls (Supplementary Table 2).

**Table 3:** Characteristics of the 14,678 study subjects

Characteristic	Hypertension	Controls	*P*
No. of subjects	8215	6463	
Age (years)	67.2 ± 12.1	55.8 ± 14.0	<0.0001
Sex (male/female, %)	59.6/40.4	51.4/48.6	<0.0001
Body mass index (kg/m^2^)	23.9 ± 3.6	22.6 ± 3.2	<0.0001
Current or former smoker (%)	35.3	37.9	0.0021
Diabetes mellitus (%)	35.3	12.1	<0.0001
Dyslipidemia (%)	69.6	51.7	<0.0001
Chronic kidney disease (%)	32.4	14.0	<0.0001
Hyperuricemia (%)	23.6	11.1	<0.0001
Systolic blood pressure (mmHg)	144 ± 24	116 ± 12	<0.0001
Diastolic blood pressure (mmHg)	81 ± 14	70 ± 10	<0.0001

Quantitative data are means ± SD and were compared between subjects with hypertension and controls with the unpaired Student's *t* test. Categorical data were compared between the two groups with Fisher's exact test. Based on Bonferroni's correction, a *P* value of <0.0050 (0.05/10) was considered statistically significant.

### Multivariable logistic regression analysis of the relation of SNPs to hypertension

The relation of the 100 SNPs identified in the EWAS for hypertension to this condition was further examined by multivariable logistic regression analysis with adjustment for age and sex (Supplementary Table 3). Nine SNPs were related (*P* < 0.01 in any one genetic model) to hypertension (Table 4), and five of these SNPs (rs150854849 of *DCLRE1C*, rs202069030 of *DUS2*, rs139012426 of *LOC100505549*, rs12229654 at 12q24.1, rs76974938 of *C21orf59*) were significantly (*P* < 1.25 × 10^−4^) associated with this condition. The minor *T* allele of rs150854849 was a risk factor for hypertension, whereas the minor *C* (rs202069030), *C* (rs139012426), *G* (rs12229654), and *T* (rs76974938) alleles of the other four SNPs were protective against this condition. The rs12229654 SNP at 12q24.1 was significantly associated with both systolic and diastolic BP as well as with hypertension.

**Table 4:** Relation of SNPs to hypertension as determined by multivariable logistic regression analysis

SNP		Dominant		Recessive		Additive 1		Additive 2	
		*P*	OR (95% CI)	*P*	OR (95% CI)	*P*	OR (95% CI)	*P*	OR (95% CI)
rs150854849	C/T (R179Q)	**1.90 × 10^−16^**	6.53 (3.79–12.41)	ND		**1.90 × 10^−16^**	6.53 (3.79–12.41)	ND	
rs2867125	G/A	0.0058	0.88 (0.80–0.96)	0.4777		0.0076	0.88 (0.80–0.97)	0.4056	
rs202069030	G/C (R51S)	**1.01 × 10^−15^**	0.15 (0.08–0.25)	ND		**1.01 × 10^−15^**	0.15 (0.08–0.25)	ND	
rs139012426	G/C (S1242T)	**7.12 × 10^−11^**	0.24 (0.15–0.38)	ND		**7.12 × 10^−11^**	0.24 (0.15–0.38)	ND	
rs75432131	G/A (H606Y)	0.0046	1.36 (1.10–1.69)	0.2511		0.0065	1.35 (1.09–1.67)	0.2473	
rs12229654	T/G	**1.13 × 10^−6^**	0.83 (0.77–0.90)	0.0132	0.82 (0.70–0.96)	**1.44 × 10^−5^**	0.84 (0.78–0.91)	0.0014	0.77 (0.65–0.90)
rs76974938	C/T (D67N)	**5.14 × 10^−5^**	0.69 (0.57–0.82)	ND		**5.14 × 10^−5^**	0.69 (0.57–0.82)	ND	
rs201633733	A/G	0.0025	2.80 (1.43–5.73)	ND		0.0025	2.80 (1.43–5.73)	ND	
rs12352313	A/G (N901S)	0.0086	0.89 (0.81–0.97)	0.0518		0.0240	0.90 (0.82–0.99)	0.0394	0.70 (0.51–0.98)

Multivariable logistic regression analysis was performed with adjustment for age and sex. Based on Bonferroni's correction, *P* values of <1.25 × 10^−4^ (0.05/400) were considered statistically significant and are shown in bold. OR, odds ratio; CI, confidence interval; ND, not determined.

### Relation of SNPs identified in the present study to systolic or diastolic BP

We next examined the relation of genotypes for the 50 SNPs isolated in the present study to systolic or diastolic BP by one-way analysis of variance (ANOVA) (Table 5). The SNP rs12229654, which was identified in the EWASs for systolic and diastolic BP and hypertension, was significantly (*P* < 0.0005) associated with both systolic and diastolic BP. The SNPs rs671, rs11066015, rs2074356, rs3782886, and rs11066280 identified in the EWASs for systolic and diastolic BP were also significantly associated with both systolic and diastolic BP. In addition, rs141569282 of *OR4F6*, rs2523638 at 6p21.3, and rs10849915 of *CCDC63* identified in the EWAS of systolic BP were significantly associated with both systolic and diastolic BP. The SNPs rs150854849, rs202069030, rs139012426, and rs76974938 identified in the EWAS for hypertension were not significantly associated with either systolic or diastolic BP, probably because of the effect of antihypertensive treatment.

**Table 5:** Relation of SNPs to systolic or diastolic blood pressure (BP)

SNP		Systolic BP (mmHg)			*P*	Diastolic BP (mmHg)			*P*
**Associated with systolic and diastolic BP and hypertension**									
rs12229654	T/G	*TT*	*TG*	*GG*		*TT*	*TG*	*GG*	
		132 ± 24	130 ± 23	128 ± 24	**1.16 × 10^−10^**	77 ± 14	75 ± 13	75 ± 13	**6.50 × 10^−12^**
**Associated with systolic and diastolic BP**									
rs671	G/A (E504K)	*GG*	*GA*	*AA*		*GG*	*GA*	*AA*	
		132 ± 24	130 ± 23	129 ± 24	**2.32 × 10^−11^**	77 ± 14	75 ± 13	75 ± 13	**5.02 × 10^−15^**
rs11066015	G/A	*GG*	*GA*	*AA*		*GG*	*GA*	*AA*	
		132 ± 24	130 ± 23	129 ± 24	**3.87 × 10^−11^**	77 ± 14	75 ± 13	75 ± 13	**7.37 × 10^−15^**
rs2074356	C/T	*CC*	*CT*	*TT*		*CC*	*CT*	*TT*	
		132 ± 24	130 ± 23	129 ± 24	**9.57 × 10^−10^**	77 ± 14	75 ± 13	74 ± 13	**1.40 × 10^−12^**
rs3782886	A/G	*AA*	*AG*	*GG*		*AA*	*AG*	*GG*	
		132 ± 24	130 ± 23	129 ± 24	**2.38 × 10^−9^**	77 ± 14	75 ± 13	75 ± 13	**4.40 × 10^−14^**
rs11066280	T/A	*TT*	*TA*	*AA*		*TT*	*TA*	*AA*	
		132 ± 24	130 ± 23	129 ± 24	**4.71 × 10^−9^**	77 ± 14	75 ± 13	75 ± 13	**9.81 × 10^−14^**
**Associated with systolic BP**									
rs78010183	A/T (T1305S)	*AA*	*AT*			*AA*	*AT*		
		132 ± 24	120 ± 16		<**1.0 × 10^−23^**	76 ± 14	74 ± 12		0.0011
rs141569282	G/A (A117T)	*GG*	*GA*			*GG*	*GA*		
		134 ± 25	121 ± 17		<**1.0 × 10^−23^**	77 ± 14	74 ± 12		**0.0002**
rs200982668	G/A (E2501K)	*GG*	*GA*			*GG*	*GA*		
		131 ± 24	120 ± 16		**2.50 × 10^−22^**	76 ± 14	75 ± 12		0.0473
rs61734696	G/T (Q137K)	*GG*	*GT*			*GG*	*GT*		
		131 ± 24	119 ± 16		**3.50 × 10^−22^**	76 ± 14	75 ± 12		0.0303
rs200787930	C/T (E1095K)	*CC*	*CT*			*CC*	*CT*		
		131 ± 24	119 ± 16		**6.50 × 10^−22^**	76 ± 14	75 ± 12		0.0640
rs139537100	C/T (R24Q)	*CC*	*CT*			*CC*	*CT*		
		131 ± 24	120 ± 16		**2.54 × 10^−21^**	76 ± 14	75 ± 12		0.0449
rs199921354	C/T (R80Q)	*CC*	*CT*			*CC*	*CT*		
		131 ± 24	120 ± 16		**4.06 × 10^−21^**	76 ± 14	75 ± 12		0.0471
rs188378669	G/T (E31*)	*GG*	*GT*			*GG*	*GT*		
		131 ± 24	120 ± 16		**1.40 × 10^−20^**	76 ± 14	75 ± 12		0.0912
rs146092501	C/T (E1386K)	*CC*	*CT*			*CC*	*CT*		
		131 ± 24	120 ± 16		**2.45 × 10^−20^**	76 ± 14	75 ± 12		0.0565
rs146879198	G/A (R340*)	*GG*	*GA*			*GG*	*GA*		
		131 ± 24	120 ± 16		**2.70 × 10^−20^**	76 ± 14	75 ± 12		0.0982
rs115287176	G/A (R277W)	*GG*	*GA*			*GG*	*GA*		
		131 ± 24	120 ± 16		**5.08 × 10^−20^**	76 ± 14	75 ± 12		0.0941
rs192210727	G/T (R580I)	*GG*	*GT*	*TT*		*GG*	*GT*	*TT*	
		131 ± 24	120 ± 16	118 ± 10	**8.83 × 10^−19^**	76 ± 14	75 ± 12	70 ± 11	0.1403
rs199576535	G/A (V341I)	*GG*	*GA*			*GG*	*GA*		
		131 ± 24	120 ± 17		**5.70 × 10^−16^**	76 ± 14	75 ± 14		0.0243
rs147284320	C/T (V503I)	*CC*	*CT*			*CC*	*CT*		
		128 ± 21	120 ± 16		**4.36 × 10^−14^**	75 ± 12	75 ± 12		0.6346
rs201459911	G/A (A1115V)	*GG*	*GA*			*GG*	*GA*		
		131 ± 24	143 ± 28		**1.16 × 10^−14^**	76 ± 14	76 ± 16		0.4868
rs199976159	G/A (G222S)	*GG*	*GA*			*GG*	*GA*		
		131 ± 24	149 ± 25		**3.98 × 10^−9^**	76 ± 14	73 ± 15		0.0627
rs2523638	G/A	*GG*	*GA*	*AA*		*GG*	*GA*	*AA*	
		130 ± 23	131 ± 24	133 ± 25	**7.24 × 10^−8^**	76 ± 13	76 ± 13	77 ± 14	**0.0002**
rs11191454	A/G	*AA*	*AG*	*GG*		*AA*	*AG*	*GG*	
		132 ± 24	130 ± 24	128 ± 22	**1.43 × 10^−7^**	77 ± 14	76 ± 13	75 ± 13	0.0032
rs12182351	T/C	*TT*	*TC*	*CC*		*TT*	*TC*	*CC*	
		130 ± 23	132 ± 25	132 ± 24	**2.43 × 10^−9^**	76 ± 13	77 ± 14	76 ± 14	0.0010
rs12413409	G/A	*GG*	*GA*	*AA*		*GG*	*GA*	*AA*	
		132 ± 24	130 ± 24	128 ± 22	**2.03 × 10^−7^**	77 ± 14	76 ± 14	75 ± 13	0.0029
rs144936999	G/C (A332P)	*GG*	*GC*			*GG*	*GC*		
		131 ± 24	154 ± 32		**4.16 × 10^−8^**	76 ± 14	76 ± 15		0.9989
rs404890	G/T	*GG*	*GT*	*TT*		*GG*	*GT*	*TT*	
		130 ± 23	132 ± 25	132 ± 24	**7.91 × 10^−9^**	76 ± 13	77 ± 14	76 ± 14	0.0018
rs130075	C/T (R102Q)	*CC*	*CT*	*TT*		*CC*	*CT*	*TT*	
		132 ± 24	129 ± 23	129 ± 22	**8.48 × 10^−8^**	76 ± 14	76 ± 13	75 ± 14	0.0152
rs11191580	T/C	*TT*	*TC*	*CC*		*TT*	*TC*	*CC*	
		132 ± 24	130 ± 24	128 ± 22	**4.44 × 10^−7^**	77 ± 14	76 ± 13	75 ± 13	0.0027
rs1058026	T/G	*TT*	*TG*	*GG*		*TT*	*TG*	*GG*	
		132 ± 24	130 ± 24	129 ± 22	**6.18 × 10^−7^**	77 ± 14	76 ± 13	76 ± 13	0.1202
rs11191548	T/C	*TT*	*TC*	*CC*		*TT*	*TC*	*CC*	
		132 ± 24	130 ± 24	128 ± 22	**6.26 × 10^−7^**	77 ± 14	76 ± 13	75 ± 13	0.0033
rs2270191	C/T (V5M)	*CC*	*CT*	*TT*		*CC*	*CT*	*TT*	
		132 ± 24	129 ± 23	129 ± 22	**4.19 × 10^−7^**	76 ± 14	76 ± 13	75 ± 14	0.0490
rs117951780	C/T (S453N)	*CC*	*CT*	*TT*		*CC*	*CT*	*TT*	
		132 ± 24	129 ± 23	129 ± 22	**5.95 × 10^−7^**	76 ± 14	76 ± 13	75 ± 14	0.0461
rs2270190	T/C	*TT*	*TC*	*CC*		*TT*	*TC*	*CC*	
		132 ± 24	129 ± 23	129 ± 22	**5.73 × 10^−7^**	76 ± 14	76 ± 13	76 ± 14	0.0360
rs17115100	G/T	*GG*	*GT*	*TT*		*GG*	*GT*	*TT*	
		132 ± 24	131 ± 24	128 ± 21	**3.37 × 10^−7^**	77 ± 14	76 ± 14	75 ± 13	0.0101
rs72655343	C/A	*CC*	*CA*			*CC*	*CA*		
		131 ± 24	149 ± 30		**4.24 × 10^−7^**	76 ± 14	77 ± 17		0.7231
rs1063646	C/T (P133L)	*CC*	*CT*	*TT*		*CC*	*CT*	*TT*	
		132 ± 24	129 ± 23	130 ± 23	**6.24 × 10^−7^**	76 ± 14	76 ± 13	76 ± 14	0.0473
rs9263739	C/T	*CC*	*CT*	*TT*		*CC*	*CT*	*TT*	
		132 ± 24	129 ± 23	130 ± 23	**6.24 × 10^−7^**	76 ± 14	76 ± 13	76 ± 14	0.0473
rs1004467	T/C	*TT*	*TC*	*CC*		*TT*	*TC*	*CC*	
		132 ± 24	131 ± 24	128 ± 21	**2.28 × 10^−6^**	77 ± 14	76 ± 14	75 ± 13	0.0208
rs139421991	G/A (R320Q)	*GG*	*GA*			*GG*	*GA*		
		131 ± 24	143 ± 28		**6.74 × 10^−7^**	76 ± 14	79 ± 18		0.0280
rs7757012	T/C	*TT*	*TC*	*CC*		*TT*	*TC*	*CC*	
		132 ± 24	129 ± 23	130 ± 23	**1.02 × 10^−6^**	76 ± 14	76 ± 13	76 ± 13	0.0526
rs142979264	C/T	*CC*	*CT*	*TT*		*CC*	*CT*	*TT*	
		132 ± 24	129 ± 23	128 ± 21	**3.71 × 10^−6^**	76 ± 14	76 ± 13	76 ± 13	0.0756
rs10849915	T/C	*TT*	*TC*	*CC*		*TT*	*TC*	*CC*	
		132 ± 24	130 ± 24	129 ± 23	**7.27 × 10^−6^**	77 ± 14	76 ± 13	75 ± 13	**7.60 × 10^−5^**
**Associated with diastolic BP**									
rs7969300	T/C (N248S)	*TT*	*TC*	*CC*		*TT*	*TC*	*CC*	
		130 ± 24	131 ± 24	133 ± 25	0.0039	76 ± 14	76 ± 14	78 ± 14	**4.59 × 10^−8^**
rs12231744	C/T (R876K)	*CC*	*CT*	*TT*		*CC*	*CT*	*TT*	
		131 ± 24	131 ± 24	133 ± 25	0.0145	76 ± 14	76 ± 14	78 ± 14	**1.44 × 10^−7^**
**Associated with hypertension**									
rs150854849	C/T (R179Q)	*CC*	*CT*			*CC*	*CT*		
		131 ± 25	133 ± 23		0.5574	76 ± 14	76 ± 10		0.7538
rs202069030	G/C (R51S)	*GG*	*GC*			*GG*	*GC*		
		131 ± 24	131 ± 24		0.9913	76 ± 14	75 ± 10		0.6866
rs139012426	G/C (S1242T)	*GG*	*GC*			*GG*	*GC*		
		131 ± 24	138 ± 26		0.1728	76 ± 14	77 ± 9		0.8428
rs76974938	C/T (D67N)	*CC*	*CT*			*CC*	*CT*		
		131 ±25	132 ±19		0.1772	76 ± 14	75 ± 11		0.0291

Data are compared among genotypes by one-way analysis of variance. Based on Bonferroni's correction, *P* values of <0.0005 (0.05/100) were considered statistically significant and are shown in bold.

### Linkage disequilibrium and haplotype analysis

Given that the six SNPs (rs12229654, rs671, rs11066015, rs2074356, rs3782886, rs11066280) found to be associated with both systolic and diastolic BP are all located at chromosomal region 12q24.12-q24.13, we examined linkage disequilibrium among these polymorphisms as well as the relation of their haplotypes to hypertension. The six SNPs were all in strong linkage disequilibrium (Supplementary Table 4). Haplotype analysis revealed that the haplotypes T (rs12229654)-G (rs671)-G (rs11066015)-C (rs2074356)-A (rs3782886)-T (rs11066280) and G (rs12229654)-A (rs671)-A (rs11066015)-T (rs2074356)-G (rs3782886)-A (rs11066280) were significantly (*P* < 9.62 × 10^−4^) associated with hypertension, with the former haplotype representing a risk factor for and the latter being protective against this condition (Supplementary Table 5).

### Relation of chromosomal loci, genes, and SNPs identified in the present study to phenotypes examined in previous GWASs

Finally, we examined the relation of the 50 SNPs of 44 genes or chromosomal loci identified in the present study to phenotypes previously probed in GWASs that are available in a public database [GWAS Catalog (National Human Genome Research Institute and European Bioinformatics Institute), http://www.ebi.ac.uk/gwas] (Supplementary Table 6). *ALDH2* [10], *HECTD4* [10], *AS3MT* [12], *CNNM2* [6, 10, 13, 14], *NT5C2* [10], and *CYP17A1* [4, 10] were previously identified as loci associated with BP in previous GWASs.

## DISCUSSION

We have now shown that rs12229654 at 12q24.1 was significantly associated with both systolic and diastolic BP as well as with hypertension and that rs671 of *ALDH2*, rs11066015 of *ACAD10*, rs2074356 and rs11066280 of *HECTD4*, and rs3782886 of *BRAP* were significantly associated with both systolic and diastolic BP. Among these loci, *ALDH2* and *HECTD4* were identified as susceptibility loci for BP in a previous GWAS [10]. The remaining SNPs at three loci (12q24.1, *ACAD10*, and *BRAP*) are newly identified as genetic determinants of systolic and diastolic BP in Japanese. We also uncovered an additional 38 and two SNPs that were associated with systolic or diastolic BP, respectively, as well as four SNPs associated with hypertension.

The six SNPs (rs12229654, rs671, rs11066015, rs2074356, rs3782886, rs11066280) significantly associated with BP are clustered at 12q24.12-q24.13 and are in linkage disequilibrium with each other. The SNP rs12229654 at 12q24.1 was previously shown to be associated with body mass index [15] and metabolic syndrome [16], with obesity and metabolic syndrome both being risk factors for elevated BP. The aldehyde dehydrogenase 2 family gene (*ALDH2*) is located at 12q24.12 (NCBI Gene, https://www.ncbi.nlm.nih.gov/gene) and is expressed in various organs and tissues, especially the liver (The Human Protein Atlas, http://www.proteinatlas.org). ALDH2 catalyze the second step of the major oxidative pathway of alcohol metabolism (NCBI Gene). *ALDH2* was previously shown to be associated with BP and hypertension [8, 10] The acyl-CoA dehydrogenase family member 10 gene (*ACAD10*) is located at 12q24.12 (NCBI Gene), is ubiquitously expressed (The Human Protein Atlas), and activates the β-oxidation of fatty acids in mitochondria (NCBI Gene). *ACAD10* has not previously been associated with BP or hypertension, although rs11066015 of this gene was shown to be related to coronary artery disease [17]. The HECT domain E3 ubiquitin protein ligase 4 gene (*HECTD4*) is located at 12q24.13 (NCBI Gene) and is broadly expressed (The Human Protein Atlas). E3 ligases accept ubiquitin from an E2 ubiquitin-conjugating enzyme in the form of a thioester and then directly transfer the ubiquitin moiety to target substrates [Universal Protein Resource, http://www.uniprot.org]. *HECTD4* was previously found to be associated with systolic and diastolic BP [10, 18]. The BRCA1 associated protein gene (*BRAP*) is located at 12q24.12 (NCBI Gene) and is ubiquitously expressed, with its expression level being especially high in the testis (The Human Protein Atlas). *BRAP* has not previously been associated with BP or hypertension, although it was found to be a susceptibility locus for myocardial infarction [19].

In recent large-scale GWASs, the MAFs of BP-associated SNPs were 5% to 50% (mean, 29.8%) and their effect sizes for systolic or diastolic BP were 0.07 to 1.13 mmHg (mean, 0.46 mmHg) and 0.06 to 0.6 mmHg (mean, 0.28 mmHg), respectively [20]. In our study, the MAFs of the six SNPs (rs12229654, rs671, rs11066015, rs2074356, rs3782886, rs11066280) associated with both systolic and diastolic BP were 22.5% to 29.3% and the differences in systolic or diastolic BP among genotypes were 3 to 4 mmHg and 2 to 3 mmHg, respectively. These SNPs were thus common variants with moderate effect sizes. We also detected an additional 38 and two SNPs associated with systolic or diastolic BP, respectively. Nineteen of these 38 SNPs associated with systolic BP had a MAF of 0.1% to 2.0% and the corresponding differences in systolic BP among (or between) genotypes were 11 to 23 mmHg; these SNPs were thus low-frequency or rare variants with large effect sizes. The remaining 19 SNPs were common variants (MAF, 11.8% to 43.1%) with differences in systolic BP among (or between) genotypes being 2 to 4 mmHg. The additional two SNPs associated with diastolic BP were common variants (MAF, 35.1% and 38.8%), with the differences in diastolic BP among genotypes being 2 mmHg.

There were several limitations to the present study: (1) Given that the results were not replicated, they will require validation in other independent populations or in other ethnic groups. (2) Given that subjects were recruited by different methods, hypertension may be a more reliable phenotype compared with BP in the present study. Therefore the relation of SNPs to systolic and diastolic BP should be interpreted carefully. (3) Given that the prevalence of hypertension was higher in the subjects recruited from the hospitals than in those from the general population, selection bias could not be excluded. (4) It is possible that the SNPs identified in the present study are in linkage disequilibrium with other polymorphisms in nearby genes that are actually responsible for the regulation of BP or the development of hypertension. (5) Four SNPs identified as susceptibility loci for hypertension were not associated with systolic or diastolic BP, possibly as a result of the effect of antihypertensive treatment. (6) The functional relevance of the identified SNPs to the pathogenesis of hypertension remains to be determined.

In conclusion, we have identified six SNPs (rs12229654 at 12q24.1, rs671 of *ALDH2*, rs11066015 of *ACAD10*, rs2074356 and rs11066280 of *HECTD4*, and rs3782886 of *BRAP*) as genetic determinants of BP. Among these loci, *ALDH2* and *HECTD4* were previously associated with BP [10]. The remaining SNPs at three loci (12q24.1, *ACAD10*, and *BRAP*) are newly identified as determinants of BP in Japanese. Determination of genotypes for these SNPs may prove informative for assessment of the genetic risk for hypertension in Japanese.

## MATERIALS AND METHODS

### Study subjects

A total of 14,678 subjects (8215 patients with hypertension and 6463 control individuals) was examined. The subjects were recruited from individuals as described previously [21].

The subjects with hypertension either had a systolic BP of ≥140 mmHg or diastolic BP of ≥90 mmHg (or both) or had taken antihypertensive medication. Individuals with severe valvular heart disease, congenital malformations of the heart or vessels, renal or endocrinologic diseases that cause secondary hypertension, or drug-induced hypertension were excluded from the study. The control individuals had a systolic BP of <140 mmHg and diastolic BP of <90 mmHg as well as no history of hypertension or of taking antihypertensive medication. Autopsy cases were excluded from controls. BP was measured at least twice with subjects having first rested in the sitting position for >5 min; the measurements were taken by a skilled physician or nurse according to the guidelines of the American Heart Association [22].

### EWASs for systolic or diastolic BP and for hypertension

Methods for collection and extraction of genomic DNA samples were described previously [21]. The EWASs were performed with the use of a HumanExome-12 v1.1 or v1.2 DNA Analysis BeadChip or Infinium Exome-24 v1.0 BeadChip (Illumina, San Diego, CA, USA). Exome array contains ~244,000 SNPs including common, low frequency, and rare variants located at whole exons. The GWAS makes use of high-throughput genotyping technologies that include up to 4.5 million markers for SNPs and copy number variations to examine their relation to clinical conditions or traits. The EWAS is a focus genotyping method that differs from the GWAS [23]. Detailed information of the exome arrays and methods of quality control were described previously [21]. A total of 41,843 SNPs passed quality control and was subjected to analysis.

### Statistical analysis

For analysis of characteristics of the study subjects, quantitative data were compared between patients with hypertension and controls with the unpaired Student's *t* test. Categorical data were compared between the two groups with Fisher's exact test. Allele frequencies were estimated by the gene counting method, and Fisher's exact test was applied to identify departure from Hardy-Weinberg equilibrium. The relation of genotype of each SNP to systolic or diastolic BP in the EWAS was analyzed with a linear regression model. Allele frequencies of SNPs were compared between subjects with hypertension and controls in the EWAS with Fisher's exact test. To compensate for multiple comparisons of genotypes with hypertension, we applied Bonferroni's correction for statistical significance of association. Given that 41,843 SNPs were analyzed, the significance level was set at *P* < 1.19 × 10^−6^ (0.05/41,843) for each EWAS. Quantile-quantile plots for *P* values of genotypes or allele frequencies in the EWASs for systolic or diastolic BP or for hypertension are shown in Supplementary Figure 2. The inflation factor (λ) was 0.95 for systolic BP, 1.05 for diastolic BP, and 1.11 for hypertension. Multivariable logistic regression analysis was performed with hypertension as a dependent variable and independent variables including age, sex (0, woman; 1, man), and genotype of each SNP. A detailed method of analysis was described previously [21]. The relation of genotypes of isolated SNPs to systolic or diastolic BP was examined with one-way ANOVA. Bonferron's correction was also applied to other analyses as indicated. Statistical tests were performed with JMP Genomics version 6.0 software (SAS Institute, Cary, NC, USA).

## SUPPLEMENTARY FIGURES AND TABLES




