# Supplementary material for: Identification of polymorphisms in 12q24.1, ACAD10, and BRAP as novel genetic determinants of blood pressure in Japanese by exome-wide association studies
Source: Oncotarget. 2017 Apr 27;8(26):43068–79. doi: 10.18632/oncotarget.17474 (PMC5522128; doi:10.18632/oncotarget.17474)
Supplement: Supplementary file 1 [file oncotarget-08-43068-s001.pdf]

Identification of polymorphisms in 12q24.1, *ACAD10*, and *BRAP* as novel genetic determinants of blood pressure in Japanese by exome-wide association studies

SUPPLEMENTARY MATERIALS

SUPPLEMENTARY FIGURES AND TABLES

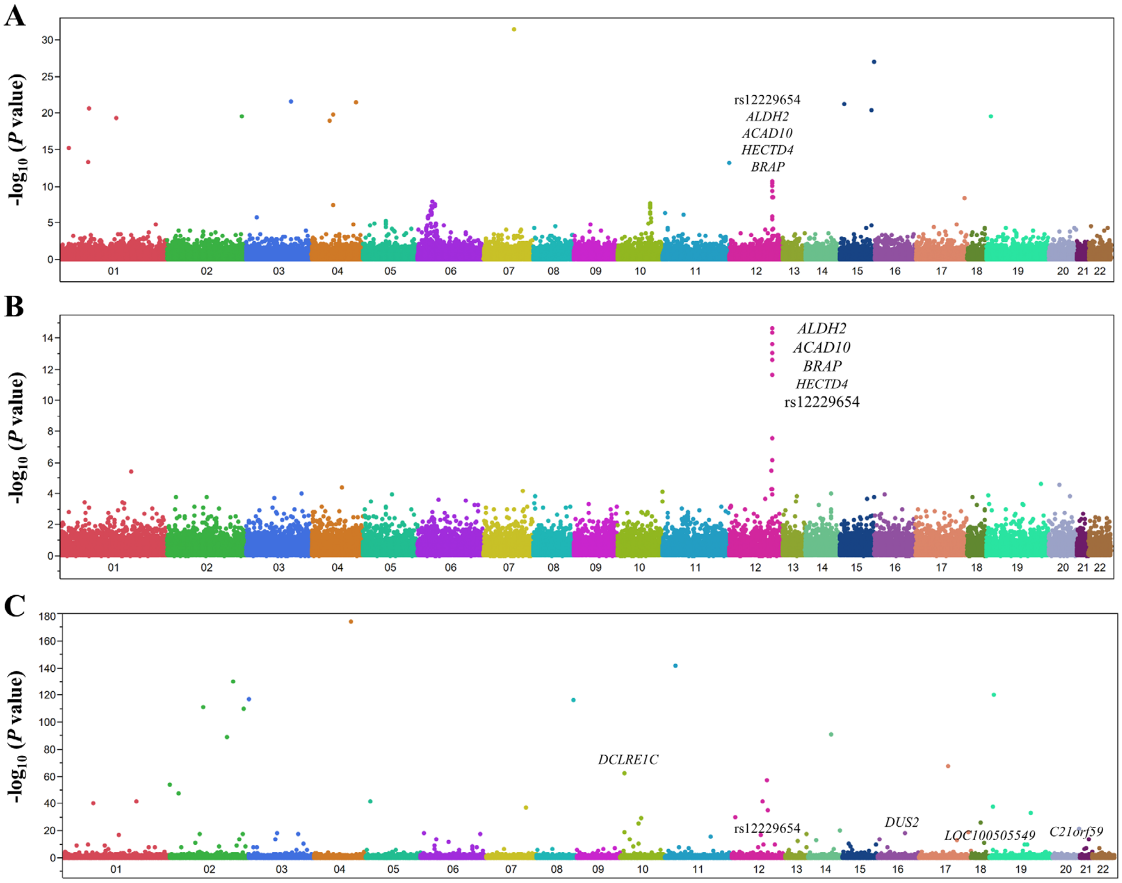

Supplementary Figure 1: Manhattan plots for *P* values in the EWASs of systolic BP (A), diastolic BP (B), and hypertension (C). The *P* values (*y*-axis) are plotted as $-\log_{10}(P)$ with respect to the physical chromosomal position of the corresponding SNPs (*x*-axis). The SNPs or genes found to be associated with both systolic and diastolic BP are indicated in (**A**) and (**B**), and those associated with hypertension are indicated in (**C**).

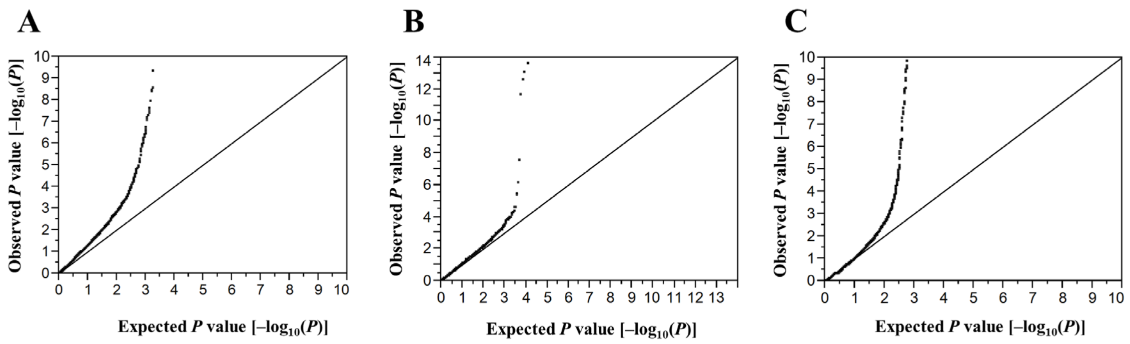

Supplementary Figure 2: Quantile-quantile plots for *P* values of allele frequencies in the EWASs of systolic BP (A), diastolic BP (B), and hypertension (C). The observed *P* values (*y*-axis) were compared with the expected *P* values (*x*-axis) under the null hypothesis, with the values being plotted as $-\log_{10}(P)$.

Supplementary Table 1: The 100 single nucleotide polymorphisms (SNPs) significantly ($P < 1.19 \times 10^{-6}$) associated with hypertension in the exome-wide association study

See Supplementary File 1

Supplementary Table 2: Genotype distributions for single nucleotide polymorphisms (SNPs) significantly ($P < 1.19 \times 10^{-6}$) associated with hypertension in the exome-wide association study

See Supplementary File 1

Supplementary Table 3: Association of SNPs with hypertension as determined by multivariable logistic regression analysis

See Supplementary File 1

Supplementary Table 4: Linkage disequilibrium of rs12229654, rs671, rs11066015, rs2074356, rs3782886, and rs11066280

	rs12229654	rs671	rs11066015	rs2074356	rs3782886	rs11066280
rs12229654		<0.0001	<0.0001	<0.0001	<0.0001	<0.0001
rs671	0.9059 (0.6155)		<0.0001	<0.0001	<0.0001	<0.0001
rs11066015	0.9071 (0.6174)	0.9964 (0.9924)		<0.0001	<0.0001	<0.0001
rs2074356	0.8034 (0.5370)	0.9950 (0.8924)	0.9911 (0.8857)		<0.0001	<0.0001
rs3782886	0.9063 (0.5656)	0.9977 (0.9140)	0.9995 (0.9170)	0.9928 (0.8154)		<0.0001
rs11066280	0.8757 (0.5336)	0.9714 (0.8755)	0.9690 (0.8709)	0.9943 (0.8266)	0.9789 (0.9483)	

Lower left data are pairwise linkage disequilibrium coefficients (*D*'), with those in parentheses being standard linkage disequilibrium coefficients (r^2). Right upper data are *P* values.

Supplementary Table 5: Relation of haplotypes of six SNPs to hypertension

Haplotype	Overall frequency	Frequency		Chi-square *P* value	Permutation *P* value
		Hypertension	Control		
T-G-G-C-A-T	0.6752	0.6942	0.6508	**7.92×10^{-11}**	**$<1.0 \times 10^{-12}$**
G-A-A-T-G-A	0.1995	0.1836	0.2200	**1.69×10^{-10}**	**$<1.0 \times 10^{-12}$**
T-A-A-T-G-A	0.0648	0.0638	0.0659	0.5494	0.566
T-G-G-C-G-A	0.0159	0.0164	0.0152	0.5008	0.489
G-A-A-C-G-A	0.0148	0.0138	0.0161	0.1817	0.200
G-G-G-C-A-T	0.0144	0.0142	0.0147	0.7686	0.770
G-A-A-C-G-T	0.0048	0.0037	0.0061	0.0149	0.014
T-A-A-C-G-A	0.0030	0.0026	0.0036	0.2487	0.282
T-G-G-C-A-A	0.0030	0.0030	0.0030	0.9725	0.998
G-A-A-T-G-T	0.0009	0.0008	0.0009	0.9350	0.942
T-G-G-T-A-A	0.0007	0.0008	0.0005	0.3438	0.357
T-A-A-C-G-T	0.0006	0.0007	0.0004	0.3800	0.460
T-G-G-C-G-T	0.0006	0.0005	0.0007	0.6531	0.673
T-A-G-T-A-A	0.0006	0.0004	0.0008	0.1908	0.223
T-A-A-T-G-T	0.0003	0.0002	0.0006	0.1699	0.230
G-G-A-C-G-T	0.0003	0.0004	0.0001	0.2864	0.379
G-G-G-C-G-T	0.0002	8.84×10^{-5}	0.0004	0.1830	0.226
G-A-G-T-G-A	0.0002	0.0004	4.86×10^{-255}	0.0781	0.034
T-G-A-C-G-A	0.0002	8.89×10^{-5}	0.0002	0.4227	0.558
T-G-G-T-G-A	5.14×10^{-5}	1.02×10^{-19}	0.0001	0.2499	0.127
T-A-G-T-G-A	5.04×10^{-5}	4.95×10^{-22}	0.0001	0.2579	0.439
G-G-A-C-G-A	5.03×10^{-5}	8.90×10^{-5}	3.43×10^{-212}	0.3781	0.766
G-G-A-T-G-A	5.02×10^{-5}	8.93×10^{-5}	2.78×10^{-176}	0.3772	0.655
G-G-A-C-A-T	5.01×10^{-5}	8.89×10^{-5}	1.64×10^{-206}	0.3781	0.431
G-A-G-T-A-A	4.28×10^{-5}	8.15×10^{-5}	9.19×10^{-66}	0.3987	0.858
G-G-G-C-G-A	3.30×10^{-8}	4.00×10^{-9}	2.48×10^{-5}	0.5978	0.219

Haplotypes consist of rs12229654 (T/G), rs671 (G/A), rs11066015 (G/A), rs2074356 (C/T), rs3782886 (A/G), and rs11066280 (T/A). Based on Bonferroni's correction, P values of $<9.62 \times 10^{-4}$ (0.05/52) were considered statistically significant and are shown in bold.

Supplementary Table 6: Relation of chromosomal loci, genes, and SNPs identified in the present study to previously examined phenotypes

See Supplementary File 1
