# Supplementary material for: Identification of polymorphisms in 12q24.1, ACAD10, and BRAP as novel genetic determinants of blood pressure in Japanese by exome-wide association studies
Source: Oncotarget. 2017 Apr 27;8(26):43068–79. doi: 10.18632/oncotarget.17474 (PMC5522128; doi:10.18632/oncotarget.17474)
Supplement: Supplementary file 2 [file oncotarget-08-43068-s002.docx]

**Supplementary Table 1.** The 100 single nucleotide polymorphisms (SNPs) significantly (*P* < 1.19 × 10^–6^) associated with hypertension in the exome-wide association study.

___________________________________________________________________________________

Gene dbSNP Nucleotide Chromosome: MAF *P* (allele) Allele

(amino acid) position (%) odds ratio

substitution^a^

___________________________________________________________________________________

*SCLT1* rs77885682 C/T (R503K) 4: 128943120 4.8 7.08 × 10^–175^ 0.98

*DNHD1* rs2344828 G/A (R697Q) 11: 6528774 12.7 2.38 × 10^–142^ 1.01

*BARD1* rs2070094 C/T (V488M) 2: 214767531 36.5 7.59 × 10^–131^ 0.95

rs11669592 A/G 19: 3318512 29.9 4.40 × 10^–121^ 0.97

rs1601875 T/C 3: 3617156 29.8 7.82 × 10^–118^ 1.07

*ZC3H3* rs3750206 A/T (Y149F) 8: 143538921 14.4 5.39 × 10^–117^ 0.99

*IL1A* rs17561 C/A (A114S) 2: 112779646 10.0 9.39 × 10^–112^ 1.05

rs12614237 T/G 2: 237280635 37.9 1.80 × 10^–110^ 0.99

*MLH3* rs28756992 T/C (S845G) 14: 75047123 0.9 8.71 × 10^–92^ 0.95

*COL3A1* rs149722210 G/A (A1045T) 2: 189006384 0.5 1.28 × 10^–89^ 0.68

*SLC4A1* rs5035 T/G (D38A) 17: 44261630 5.2 3.69 × 10^–68^ 1.00

*DCLRE1C* rs150854849 C/T (R179Q) 10: 14934704 2.4 9.18 × 10^–63^ 21.69

*TMTC2* rs9651971 G/A 12: 82845502 13.6 8.27 × 10^–58^ 1.06

rs2867125 G/A 2: 622827 9.4 8.58 × 10^–55^ 0.90

*FAM179A* rs80120243 A/G (N224S) 2: 29003523 22.7 2.06 × 10^–48^ 0.99

*PAPPA2* rs191775270 G/A (V584M) 1: 176595354 0.1 2.57 × 10^–42^ 1.51

*FBXL7* rs257747 C/A 5: 15817857 42.7 5.00 × 10^–42^ 1.01

*RPSAP52* rs7134682 G/T 12: 65774371 41.8 5.43 × 10^–42^ 0.97

*PODN* rs17107806 G/C (A10P) 1: 53066863 11.7 2.87 × 10^–41^ 1.01

*ABHD17A* rs4807160 C/T (E144K) 19: 1880951 32.4 3.26 × 10^–38^ 0.98

*AKR1B10* rs3735042 T/C (M286T) 7: 134538966 8.3 1.85 × 10^–37^ 1.01

*TMTC3* rs78527125 C/G (L113V) 12: 88153438 2.6 1.87 × 10^–35^ 1.06

*ZNF225* rs62623665 G/A (R352H) 19: 44131669 0.2 9.03 × 10^–34^ 1.20

*VWF* rs216902 A/G 12: 5996221 17.9 2.69 × 10^–30^ 0.96

*CDH23* rs143993990 G/A (V1807M) 10: 71784337 0.3 3.30 × 10^–30^ 1.33

*SLC14A1* rs11877062 T/C (W4R) 18: 45727281 46.3 1.33 × 10^–26^ 1.00

*CTNNA3* rs7895803 G/A 10: 66134723 39.2 4.05 × 10^–26^ 1.02

*RTEL1* rs3208008 A/C (Q1042H) 20: 63694757 38.5 3.98 × 10^–22^ 1.03

rs11626651 C/T 14: 101717022 45.7 6.70 × 10^–21^ 1.01

*MEIG1* rs7919322 G/A 10: 14961279 41.3 7.80 × 10^–20^ 0.98

*RNF213* rs61740658 A/G (E3915G) 17: 80363784 6.6 1.48 × 10^–19^ 1.04

rs1033423 T/C 6: 11821770 4.8 4.47 × 10^–19^ 1.00

*DUS2* rs202069030 G/C (R51S) 16: 68023050 0.4 7.76 × 10^–19^ 0.14

*FRMD4B* rs182546659 T/C (M779V) 3: 69181415 0.4 8.03 × 10^–19^ 1.22

*SYNE1* rs117184249 C/T (G2297R) 6: 152401278 2.1 2.12 × 10^–18^ 0.96

rs7333181 G/A 13: 111568950 2.5 2.58 × 10^–18^ 0.94

*UGT1A1* rs34993780 T/G (Y486D) 2: 233772413 0.2 4.38 × 10^–18^ 0.81

*AFF3* rs117712488 G/T (H889Q) 2: 99582924 2.6 5.50 × 10^–18^ 0.98

rs6774273 C/T 3: 144250723 34.5 6.38 × 10^–18^ 1.00

*THEM5* rs6587625 C/T (G197D) 1: 151847848 32.5 1.62 × 10^–17^ 1.01

*STAT6* rs118014438 C/T (V159I) 12: 57105475 1.2 2.17 × 10^–17^ 0.84

*TENM4* rs3812723 C/T (V396I) 11: 78863031 1.7 4.73 × 10^–16^ 0.88

*NARFL* rs139320158 C/A (R177M) 16: 732361 1.1 1.32 × 10^–14^ 1.18

*DAW1* rs10191097 T/G 2: 227911955 29.1 1.66 × 10^–14^ 0.96

*C6orf48* rs10456397 A/T 6: 31836237 0.2 1.92 × 10^–14^ 1.02

rs9834596 A/G 3: 65048746 14.5 2.16 × 10^–14^ 1.06

*UBASH3A* rs2277800 C/T (L28F) 21: 42404027 12.4 2.27 × 10^–14^ 0.97

rs2769214 A/G 10: 32226165 32.3 3.46 × 10^–14^ 0.99

rs1952896 T/C 14: 31552232 28.5 8.40 × 10^–14^ 1.00

*APOH* rs4581 A/C (L266V) 17: 66214639 22.3 1.76 × 10^–13^ 0.96

rs9540337 A/C 13: 65036276 21.2 2.37 × 10^–13^ 1.00

*PTK7* rs78949718 G/A (R269H) 6: 43130655 0.1 1.12 × 10^–12^ 1.09

rs7589293 T/C 2: 81356433 43.7 1.06 × 10^–11^ 1.01

*LOC100505549* rs139012426 G/C (S1242T) 18: 57648519 0.4 1.15 × 10^–11^ 0.25

*HNRNPH3* rs2273903 A/G (N148S) 10: 68339191 1.2 2.55 × 10^–11^ 0.98

*ACTRT3* rs34273285 G/C (V251L) 3: 169767800 0.5 3.51 × 10^–11^ 0.82

*MGA* rs138712718 T/G (I7M) 15: 41668915 3.4 4.07 × 10^–11^ 1.08

*ADAMTS17* rs4246302 A/G 15: 100147762 30.5 1.41 × 10^–10^ 1.00

*TGFB1* rs148087680 C/T (R75Q) 19: 41355004 0.6 1.53 × 10^–10^ 1.28

*IRAK3* rs140228894 T/C (L220P) 12: 66228325 0.2 2.44 × 10^–10^ 1.24

*CAPN12* rs75432131 G/A (H606Y) 19: 38734204 1.5 3.01 × 10^–10^ 1.27

rs12229654 T/G 12: 110976657 22.5 3.27 × 10^–10^ 0.84

*PPT1* rs1800205 A/G (I134T) 1: 40091361 13.1 3.49 × 10^–10^ 1.03

*ANKZF1* rs57075420 C/T (R297C) 2: 219233784 1.8 4.56 × 10^–10^ 0.95

*PKN2* rs786906 T/C 1: 88805891 46.8 8.01 × 10^–10^ 0.99

*UBXN10* rs116369009 G/A (E152K) 1: 20191015 0.2 8.48 × 10^–10^ 1.12

rs2516399 T/C 6: 31513522 17.1 1.12 × 10^–9^ 0.99

*ESPNL* rs184614603 G/A (R913Q) 2: 238131452 0.6 2.29 × 10^–9^ 1.00

*PLA2G4D* rs11635685 G/C (R275P) 15: 42081612 44.8 2.31 × 10^–9^ 1.01

*ZFAND4* rs41301625 T/C 10: 45615591 11.7 2.69 × 10^–9^ 1.01

*MYO3B* rs13019430 G/A 2: 170362545 39.7 3.52 × 10^–9^ 1.00

*LAMA2* rs184127828 A/C (E2796D) 6: 129503121 0.6 4.13 × 10^–9^ 1.09

*FBLN2* rs2290189 C/T 3: 13636446 5.9 9.57 × 10^–9^ 0.94

*OR9K2* rs7305779 A/C (E103A) 12: 55130076 9.8 1.19 × 10^–8^ 0.93

*ST5* rs3794153 C/G (N316K) 11: 8730342 40.3 1.30 × 10^–8^ 1.01

*SOX1* rs139160172 G/A (G139S) 13: 112068073 2.8 1.73 × 10^–8^ 1.02

*TGFBR2* rs1808602 A/G 3: 30648932 38.1 1.81 × 10^–8^ 0.98

rs4896780 A/C 6: 145196271 49.7 2.67 × 10^–8^ 0.97

rs947211 G/A 1: 205783537 49.2 3.39 × 10^–8^ 1.01

rs9927623 A/C 16: 8254436 41.8 3.44 × 10^–8^ 0.96

*GPATCH2* rs35737297 T/C (M474V) 1: 217431312 4.7 3.54 × 10^–8^ 1.04

*LTBP1* rs41464348 C/T 2: 33302232 33.2 3.56 × 10^–8^ 0.96

*CRY2* rs11605924 A/C 11: 45851540 18.8 4.84 × 10^–8^ 1.05

*ERG* rs7275212 A/T 21: 38480628 8.4 4.98 × 10^–8^ 0.98

*ADAMTS13* rs142060916 C/T (S1258L) 9: 133457958 0.1 7.90 × 10^–8^ 1.05

*TFIP11* rs150843922 T/C (K703R) 22: 26494189 0.4 1.54 × 10^–7^ 1.24

rs11166240 T/C 1: 99406200 24.9 1.79 × 10^–7^ 0.98

*C9orf43* rs78738447 G/A 9: 113428964 2.2 4.39 × 10^–7^ 1.03

*C21orf59* rs76974938 C/T (D67N) 21: 32609946 2.4 4.45 × 10^–7^ 1.54

*ABCA13* rs141576983 G/T 7: 48412584 2.3 4.91 × 10^–7^ 0.97

SNTG1 rs16914781 A/G 8: 50374922 20.8 5.15 × 10^–7^ 1.03

rs3869115 G/C 6: 31236917 21.0 5.39 × 10^–7^ 0.93

rs7157338 G/A 14: 82610928 49.9 5.49 × 10^–7^ 1.02

*LAMA2* rs118147866 C/T (R826W) 6: 129280086 0.2 6.70 × 10^–7^ 1.27

*MYOM2* rs201633733 A/G 8: 2129125 0.2 7.78 × 10^–7^ 1.68

*CUZD1* rs35299879 C/A (E43D) 10: 122841282 4.2 8.72 × 10^–7^ 0.98

*AP3S1* rs26534 G/A 5: 115840004 45.5 8.79 × 10^–7^ 1.01

*KANK1* rs12352313 A/G (N901S) 9: 730054 10.3 8.79 × 10^–7^ 0.94

*KLHL24* rs116961268 A/C (D197A) 3: 183650946 0.4 9.05 × 10^–7^ 1.17

*LCA5* rs185347145 G/A (P548S) 6: 79487456 0.7 1.14 × 10^–6^ 0.98

___________________________________________________________________________________

Allele frequencies were analyzed with Fisher’s exact test. ^a^Major allele/minor allele. MAF, minor allele frequency.

**Supplementary Table 2.** Genotype distributions for single nucleotide polymorphisms (SNPs) significantly (*P* < 1.19 × 10^–6^) associated with hypertension in the exome-wide association study.

____________________________________________________________________________________________________________

SNP Hypertension H-W *P* Control H-W *P*

____________________________________________________________________________________________________________

rs77885682 C/T (R503K) *CC* *CT* *TT* *CC* *CT* *TT*

7448 (90.66) 741 (9.02) 26 (0.32) 0.1164 5843 (90.41) 602 (9.31) 18 (0.28) 0.5076

rs2344828 G/A (R697Q) *GG* *GA* *AA* *GG* *GA* *AA*

6244 (76.02) 1823 (22.19) 147 (1.79) 0.3019 4918 (76.10) 1444 (22.34) 101 (1.56) 0.7369

rs2070094 C/T (V488M) *CC* *CT* *TT* *CC* *CT* *TT*

3376 (41.10) 3746 (45.61) 1092 (13.29) 0.3037 2528 (39.12) 3059 (47.33) 876 (13.55) 0.3119

rs11669592 A/G *AA* *AG* *GG* *AA* *AG* *GG*

4069 (49.55) 3425 (41.71) 718 (8.74) 0.9578 3136 (48.53) 2752 (42.59) 574 (8.88) 0.4085

rs1601875 T/C *TT* *TC* *CC* *TT* *TC* *CC*

3983 (48.49) 3458 (42.09) 774 (9.42) 0.5493 3260 (50.45) 2648 (40.98) 554 (8.57) 0.6293

rs3750206 A/T (Y149F) *AA* *AT* *TT* *AA* *AT* *TT*

6469 (78.79) 1618 (19.70) 124 (1.51) 0.0549 5058 (78.29) 1324 (20.49) 79 (1.22) 0.5004

rs17561 C/A (A114S) *CC* *CA* *AA* *CC* *CA* *AA*

6644 (80.89) 1481 (18.03) 89 (1.08) 0.5042 5286 (81.79) 1103 (17.07) 74 (1.14) 0.0638

rs12614237 T/G *TT* *TG* *GG* *TT* *TG* *GG*

3176 (38.66) 3867 (47.07) 1172 (14.27) 0.9439 2496 (38.62) 3018 (46.70) 949 (14.68) 0.4600

rs28756992 T/C (S845G) *TT* *TC* *CC* *TT* *TC* *CC*

8081 (98.37) 132 (1.61) 2 (0.02) 0.1073 6352 (98.28) 110 (1.70) 1 (0.02) 0.3844

rs149722210 G/A (A1045T) *GG* *GA* *AA* *GG* *GA* *AA*

8148 (99.22) 64 (0.78) 0 (0) 1.0000 6388 (98.86) 74 (1.14) 0 (0) 1.0000

rs5035 T/G (D38A) *TT* *TG* *GG* *TT* *TG* *GG*

7393 (89.99) 801 (9.75) 21 (0.26) 1.0000 5821 (90.07) 623 (9.64) 19 (0.29) 0.6067

rs150854849 C/T (R179Q) *CC* *CT* *TT* *CC* *CT* *TT*

5964 (95.56) 277 (4.44) 0 (0) 0.0769 5728 (99.79) 12 (0.21) 0 (0) 1.0000

rs9651971 G/A *GG* *GA* *AA* *GG* *GA* *AA*

6101 (74.28) 1959 (23.85) 154 (1.87) 0.8529 4890 (75.66) 1456 (22.53) 117 (1.81) 0.4767

rs2867125 G/A *GG* *GA* *AA* *GG* *GA* *AA*

6812 (82.92) 1321 (16.08) 82 (1.00) 0.0513 5252 (81.26) 1142 (17.67) 69 (1.07) 0.4428

rs80120243 A/G (N224S) *AA* *AG* *GG* *AA* *AG* *GG*

4947 (60.22) 2829 (34.44) 439 (5.34) 0.1954 3872 (59.91) 2235 (34.58) 356 (5.51) 0.1575

rs191775270 G/A (V584M) *GG* *GA* *AA* *GG* *GA* *AA*

8190 (99.70) 25 (0.30) 0 (0) 1.0000 6450 (99.80) 13 (0.20) 0 (0) 1.0000

rs257747 C/A *CC* *CA* *AA* *CC* *CA* *AA*

2677 (32.60) 4013 (48.87) 1522 (18.53) 0.7871 2120 (32.80) 3151 (48.76) 1192 (18.44) 0.7222

rs7134682 G/T *GG* *GT* *TT* *GG* *GT* *TT*

2846 (34.65) 3922 (47.74) 1447 (17.61) 0.1336 2162 (33.45) 3147 (48.69) 1154 (17.86) 0.8784

rs17107806 G/C (A10P) *GG* *GC* *CC* *GG* *GC* *CC*

6411 (78.07) 1694 (20.63) 107 (1.30) 0.7470 5054 (78.20) 1324 (20.49) 85 (1.31) 0.9515

rs4807160 C/T (E144K) *CC* *CT* *TT* *CC* *CT* *TT*

3813 (46.54) 3488 (42.58) 891 (10.88) 0.0276 2893 (45.02) 2875 (44.74) 658 (10.24) 0.1559

rs3735042 T/C (M286T) *TT* *TC* *CC* *TT* *TC* *CC*

6873 (83.70) 1293 (15.74) 46 (0.56) 0.0858 5437 (84.13) 973 (15.05) 53 (0.82) 0.1932

rs78527125 C/G (L113V) *CC* *CG* *GG* *CC* *CG* *GG*

7788 (94.84) 418 (5.09) 6 (0.07) 0.8269 6146 (95.11) 312 (4.83) 4 (0.06) 0.7996

rs62623665 G/A (R352H) *GG* *GA* *AA* *GG* *GA* *AA*

8185 (99.65) 29 (0.35) 0 (0) 1.0000 6444 (99.71) 19 (0.29) 0 (0) 1.0000

rs216902 A/G *AA* *AG* *GG* *AA* *AG* *GG*

5594 (68.10) 2348 (28.59) 272 (3.31) 0.1832 4338 (67.12) 1902 (29.43) 223 (3.45) 0.4265

rs143993990 G/A (V1807M) *GG* *GA* *AA* *GG* *GA* *AA*

8154 (99.26) 61 (0.74) 0 (0) 1.0000 6427 (99.44) 36 (0.56) 0 (0) 1.0000

rs11877062 T/C (W4R) *TT* *TC* *CC* *TT* *TC* *CC*

2359 (28.74) 4085 (49.78) 1763 (21.48) 0.9646 1861 (28.79) 3214 (49.73) 1388 (21.48) 1.0000

rs7895803 G/A *GG* *GA* *AA* *GG* *GA* *AA*

3039 (37.00) 3862 (47.02) 1312 (15.98) 0.1458 2380 (36.83) 3133 (48.48) 949 (14.69) 0.1165

rs3208008 A/C (Q1042H) *AA* *AC* *CC* *AA* *AC* *CC*

3081 (37.52) 3904 (47.55) 1226 (14.93) 0.8707 2488 (38.56) 3014 (46.72) 950 (14.72) 0.4598

rs11626651 C/T *CC* *CT* *TT* *CC* *CT* *TT*

2439 (29.70) 4052 (49.33) 1722 (20.97) 0.6090 1940 (30.03) 3186 (49.31) 1335 (20.66) 0.6880

rs7919322 G/A *GG* *GA* *AA* *GG* *GA* *AA*

2862 (34.84) 3941 (47.98) 1411 (17.18) 0.3868 2194 (33.95) 3164 (48.95) 1105 (17.10) 0.5559

rs61740658 A/G (E3915G) *AA* *AG* *GG* *AA* *AG* *GG*

7150 (87.09) 1022 (12.45) 38 (0.46) 0.7907 5663 (87.62) 768 (11.88) 32 (0.50) 0.3003

rs1033423 T/C *TT* *TC* *CC* *TT* *TC* *CC*

7439 (90.60) 755 (9.19) 17 (0.21) 0.7181 5865 (90.75) 576 (8.91) 22 (0.34) 0.0562

rs202069030 G/C (R51S) *GG* *GC* *CC* *GG* *GC* *CC*

8144 (99.79) 17 (0.21) 0 (0) 1.0000 6315 (98.53) 94 (1.47) 0 (0) 1.0000

rs182546659 T/C (M779V) *TT* *TC* *CC* *TT* *TC* *CC*

8129 (99.11) 73 (0.89) 0 (0) 1.0000 6414 (99.27) 47 (0.73) 0 (0) 1.0000

rs117184249 C/T (G2297R) *CC* *CT* *TT* *CC* *CT* *TT*

7850 (95.85) 336 (4.10) 4 (0.05) 0.7843 6163 (95.64) 279 (4.33) 2 (0.03) 0.7715

rs7333181 G/A *GG* *GA* *AA* *GG* *GA* *AA*

7811 (95.09) 398 (4.85) 5 (0.06) 1.0000 6127 (94.80) 330 (5.11) 6 (0.09) 0.4615

rs34993780 T/G (Y486D) *TT* *TG* *GG* *TT* *TG* *GG*

8179 (99.62) 30 (0.37) 1 (0.01) 0.0298 6435 (99.57) 27 (0.42) 1 (0.01) 0.0310

rs117712488 G/T (H889Q) *GG* *GT* *TT* *GG* *GT* *TT*

7797 (94.91) 410 (4.99) 8 (0.10) 0.2690 6123 (94.74) 338 (5.23) 2 (0.03) 0.3300

rs6774273 C/T *CC* *CT* *TT* *CC* *CT* *TT*

3539 (43.09) 3678 (44.78) 996 (12.13) 0.3930 2794 (43.23) 2882 (44.59) 787 (12.18) 0.2955

rs6587625 C/T (G197D) *CC* *CT* *TT* *CC* *CT* *TT*

3743 (45.57) 3579 (43.57) 892 (10.86) 0.4077 2949 (45.63) 2843 (43.99) 671 (10.38) 0.7332

rs118014438 C/T (V159I) *CC* *CT* *TT* *CC* *CT* *TT*

8029 (97.74) 186 (2.26) 0 (0) 0.6285 6290 (97.32) 173 (2.68) 0 (0) 0.6318

rs3812723 C/T (V396I) *CC* *CT* *TT* *CC* *CT* *TT*

7949 (96.76) 263 (3.20) 3 (0.04) 0.4840 6225 (96.32) 237 (3.67) 1 (0.01) 0.7274

rs139320158 C/A (R177M) *CC* *CA* *AA* *CC* *CA* *AA*

8024 (97.68) 191 (2.32) 0 (0) 0.6302 6335 (98.02) 128 (1.98) 0 (0) 1.0000

rs10191097 T/G *TT* *TG* *GG* *TT* *TG* *GG*

4170 (50.77) 3367 (40.99) 677 (8.24) 0.9570 3205 (49.59) 2699 (41.76) 559 (8.65) 0.8110

rs10456397 A/T *AA* *AT* *TT* *AA* *AT* *TT*

8183 (99.62) 31 (0.38) 0 (0) 1.0000 6439 (99.63) 24 (0.37) 0 (0) 1.0000

rs9834596 A/G *AA* *AG* *GG* *AA* *AG* *GG*

5961 (72.56) 2061 (25.09) 193 (2.35) 0.3386 4785 (74.04) 1531 (23.69) 147 (2.27) 0.0647

rs2277800 C/T (L28F) *CC* *CT* *TT* *CC* *CT* *TT*

6316 (77.06) 1762 (21.50) 118 (1.44) 0.7568 4930 (76.38) 1435 (22.23) 90 (1.39) 0.2322

rs2769214 A/G *AA* *AG* *GG* *AA* *AG* *GG*

3754 (45.70) 3610 (43.95) 850 (10.35) 0.6867 2944 (45.55) 2832 (43.82) 687 (10.63) 0.8873

rs1952896 T/C *TT* *TC* *CC* *TT* *TC* *CC*

4266 (51.94) 3229 (39.31) 719 (8.75) 0.0026 3345 (51.76) 2553 (39.50) 565 (8.74) 0.0146

rs4581 A/C (L266V) *AA* *AC* *CC* *AA* *AC* *CC*

5005 (60.93) 2810 (34.21) 399 (4.86) 0.8469 3881 (60.05) 2243 (34.71) 339 (5.24) 0.5222

rs9540337 A/C *AA* *AC* *CC* *AA* *AC* *CC*

5104 (62.14) 2742 (33.38) 368 (4.48) 1.0000 4029 (62.35) 2120 (32.81) 313 (4.84) 0.1184

rs78949718 G/A (R269H) *GG* *GA* *AA* *GG* *GA* *AA*

8197 (99.78) 18 (0.22) 0 (0) 1.0000 6450 (99.80) 13 (0.20) 0 (0) 1.0000

rs7589293 T/C *TT* *TC* *CC* *TT* *TC* *CC*

2626 (31.98) 3983 (48.51) 1602 (19.51) 0.1935 2102 (32.52) 3092 (47.84) 1269 (19.64) 0.0296

rs139012426 G/C (S1242T) *GG* *GC* *CC* *GG* *GC* *CC*

7955 (99.66) 27 (0.34) 0 (0) 1.0000 6266 (98.66) 85 (1.34) 0 (0) 1.0000

rs2273903 A/G (N148S) *AA* *AG* *GG* *AA* *AG* *GG*

8017 (97.59) 195 (2.37) 3 (0.04) 0.1235 6301 (97.49) 162 (2.51) 0 (0) 0.6275

rs34273285 G/C (V251L) *GG* *GC* *CC* *GG* *GC* *CC*

8137 (99.05) 78 (0.95) 0 (0) 1.0000 6388 (98.84) 75 (1.16) 0 (0) 1.0000

rs138712718 T/G (I7M) *TT* *TG* *GG* *TT* *TG* *GG*

7631 (92.89) 574 (6.99) 10 (0.12) 1.0000 6041 (93.49) 410 (6.34) 11 (0.17) 0.1716

rs4246302 A/G *AA* *AG* *GG* *AA* *AG* *GG*

3933 (47.89) 3538 (43.08) 742 (9.03) 0.1850 3090 (47.83) 2791 (43.21) 579 (8.96) 0.1594

rs148087680 C/T (R75Q) *CC* *CT* *TT* *CC* *CT* *TT*

8103 (98.64) 112 (1.36) 0 (0) 1.0000 6394 (98.93) 69 (1.07) 0 (0) 1.0000

rs140228894 T/C (L220P) *TT* *TC* *CC* *TT* *TC* *CC*

8184 (99.64) 30 (0.36) 0 (0) 1.0000 6444 (99.71) 19 (0.29) 0 (0) 1.0000

rs75432131 G/A (H606Y) *GG* *GA* *AA* *GG* *GA* *AA*

7949 (96.76) 261 (3.18) 5 (0.06) 0.0722 6295 (97.40) 167 (2.58) 1 (0.02) 1.0000

rs12229654 T/G *TT* *TG* *GG* *TT* *TG* *GG*

5104 (62.15) 2728 (33.22) 380 (4.63) 0.5308 3708 (57.39) 2361 (36.54) 392 (6.07) 0.5431

rs1800205 A/G (I134T) *AA* *AG* *GG* *AA* *AG* *GG*

6189 (75.36) 1875 (22.83) 149 (1.81) 0.5972 4900 (75.82) 1465 (22.67) 98 (1.51) 0.3740

rs57075420 C/T (R297C) *CC* *CT* *TT* *CC* *CT* *TT*

7933 (96.57) 278 (3.38) 4 (0.05) 0.3164 6229 (96.38) 232 (3.59) 2 (0.03) 1.0000

rs786906 T/C *TT* *TC* *CC* *TT* *TC* *CC*

2327 (28.34) 4098 (49.92) 1785 (21.74) 0.8246 1842 (28.51) 3165 (48.99) 1454 (22.50) 0.1776

rs116369009 G/A (E152K) *GG* *GA* *AA* *GG* *GA* *AA*

8188 (99.67) 27 (0.33) 0 (0) 1.0000 6444 (99.71) 19 (0.29) 0 (0) 1.0000

rs2516399 T/C *TT* *TC* *CC* *TT* *TC* *CC*

5632 (68.56) 2354 (28.65) 229 (2.79) 0.3922 4442 (68.73) 1819 (28.15) 202 (3.12) 0.3368

rs184614603 G/A (R913Q) *GG* *GA* *AA* *GG* *GA* *AA*

8086 (98.68) 108 (1.32) 0 (0) 1.0000 6362 (98.68) 85 (1.32) 0 (0) 1.0000

rs11635685 G/C (R275P) *GG* *GC* *CC* *GG* *GC* *CC*

2518 (30.65) 4025 (49.00) 1672 (20.35) 0.3845 1962 (30.36) 3234 (50.04) 1267 (19.60) 0.3263

rs41301625 T/C *TT* *TC* *CC* *TT* *TC* *CC*

6412 (78.37) 1636 (19.99) 134 (1.64) 0.0154 5041 (78.33) 1311 (20.37) 84 (1.30) 0.9512

rs13019430 G/A *GG* *GA* *AA* *GG* *GA* *AA*

3033 (36.92) 3841 (46.76) 1341 (16.32) 0.0340 2369 (36.65) 3056 (47.29) 1038 (16.06) 0.3235

rs184127828 A/C (E2796D) *AA* *AC* *CC* *AA* *AC* *CC*

8111 (98.74) 103 (1.25) 1 (0.01) 0.2843 6387 (98.92) 76 (1.18) 0 (0) 1.0000

rs2290189 C/T *CC* *CT* *TT* *CC* *CT* *TT*

7293 (88.78) 897 (10.92) 25 (0.30) 0.7601 5700 (88.20) 737 (11.40) 26 (0.40) 0.6637

rs7305779 A/C (E103A) *AA* *AC* *CC* *AA* *AC* *CC*

6738 (82.02) 1393 (16.96) 84 (1.02) 0.1997 5221 (80.78) 1172 (18.14) 70 (1.08) 0.6330

rs3794153 C/G (N316K) *CC* *CG* *GG* *CC* *CG* *GG*

2922 (35.57) 3941 (47.97) 1352 (16.46) 0.7140 2319 (35.89) 3086 (47.75) 1057 (16.36) 0.5694

rs139160172 G/A (G139S) *GG* *GA* *AA* *GG* *GA* *AA*

7730 (94.42) 451 (5.51) 6 (0.07) 1.0000 6100 (94.55) 346 (5.36) 6 (0.09) 0.6393

rs1808602 A/G *AA* *AG* *GG* *AA* *AG* *GG*

3191 (38.85) 3821 (46.52) 1202 (14.63) 0.2915 2465 (38.15) 3045 (47.12) 952 (14.73) 0.8128

rs4896780 A/C *AA* *AC* *CC* *AA* *AC* *CC*

2169 (26.42) 3955 (48.18) 2085 (25.40) 0.0010 1664 (25.78) 3108 (48.15) 1683 (26.07) 0.0030

rs947211 G/A *GG* *GA* *AA* *GG* *GA* *AA*

2175 (26.47) 3984 (48.50) 2056 (25.03) 0.0071 1711 (26.47) 3152 (48.77) 1600 (24.76) 0.0522

rs9927623 A/C *AA* *AC* *CC* *AA* *AC* *CC*

2858 (34.79) 3898 (47.45) 1459 (17.76) 0.0408 2136 (33.06) 3175 (49.13) 1151 (17.81) 0.6466

rs35737297 T/C (M474V) *TT* *TC* *CC* *TT* *TC* *CC*

7443 (90.65) 743 (9.05) 25 (0.30) 0.1504 5875 (90.92) 572 (8.85) 15 (0.23) 0.7784

rs41464348 C/T *CC* *CT* *TT* *CC* *CT* *TT*

3729 (45.41) 3559 (43.34) 924 (11.25) 0.0894 2858 (44.22) 2838 (43.91) 767 (11.87) 0.1265

rs11605924 A/C *AA* *AC* *CC* *AA* *AC* *CC*

5338 (65.28) 2529 (30.93) 310 (3.79) 0.6187 4275 (66.24) 1961 (30.38) 218 (3.38) 0.7419

rs7275212 A/T *AA* *AT* *TT* *AA* *AT* *TT*

6916 (84.22) 1229 (14.97) 67 (0.81) 0.1280 5416 (83.80) 1000 (15.47) 47 (0.73) 0.8726

rs142060916 C/T (S1258L) *CC* *CT* *TT* *CC* *CT* *TT*

8190 (99.71) 24 (0.29) 0 (0) 1.0000 6445 (99.72) 18 (0.28) 0 (0) 1.0000

rs150843922 T/C (K703R) *TT* *TC* *CC* *TT* *TC* *CC*

8144 (99.14) 71 (0.86) 0 (0) 1.0000 6418 (99.30) 45 (0.70) 0 (0) 1.0000

rs11166240 T/C *TT* *TC* *CC* *TT* *TC* *CC*

4650 (56.64) 3055 (37.21) 505 (6.15) 0.9057 3647 (56.43) 2389 (36.96) 427 (6.61) 0.1856

rs78738447 G/A *GG* *GA* *AA* *GG* *GA* *AA*

7858 (95.66) 351 (4.27) 6 (0.07) 0.2980 6192 (95.81) 265 (4.10) 6 (0.09) 0.0754

rs76974938 C/T (D67N) *CC* *CT* *TT* *CC* *CT* *TT*

5947 (94.20) 366 (5.80) 0 (0) 0.0108 5725 (96.19) 227 (3.81) 0 (0) 0.2798

rs141576983 G/T *GG* *GT* *TT* *GG* *GT* *TT*

7843 (95.47) 366 (4.46) 6 (0.07) 0.4530 6162 (95.34) 297 (4.60) 4 (0.06) 0.7833

rs16914781 A/G *AA* *AG* *GG* *AA* *AG* *GG*

5165 (62.87) 2646 (32.21) 404 (4.92) 0.0071 4078 (63.10) 2124 (32.86) 261 (4.04) 0.4681

rs3869115 G/C *GG* *GC* *CC* *GG* *GC* *CC*

5232 (63.69) 2613 (31.81) 370 (4.50) 0.0617 3974 (61.49) 2187 (33.84) 302 (4.67) 0.9707

rs7157338 G/A *GG* *GA* *AA* *GG* *GA* *AA*

2048 (24.93) 4090 (49.79) 2076 (25.28) 0.7076 1654 (25.59) 3201 (49.54) 1607 (24.87) 0.4555

rs118147866 C/T (R826W) *CC* *CT* *TT* *CC* *CT* *TT*

8173 (99.49) 42 (0.51) 0 (0) 1.0000 6437 (99.60) 26 (0.40) 0 (0) 1.0000

rs201633733 A/G *AA* *AG* *GG* *AA* *AG* *GG*

8183 (99.61) 32 (0.39) 0 (0) 1.0000 6448 (99.78) 13 (0.20) 1 (0.02) 0.0081

rs35299879 C/A (E43D) *CC* *CA* *AA* *CC* *CA* *AA*

7544 (91.83) 654 (7.96) 17 (0.21) 0.4895 5929 (91.74) 517 (8.00) 17 (0.26) 0.1243

rs26534 G/A *GG* *GA* *AA* *GG* *GA* *AA*

2384 (29.03) 4186 (50.97) 1642 (20.00) 0.0119 1973 (30.53) 3132 (48.46) 1358 (21.01) 0.0789

rs12352313 A/G (N901S) *AA* *AG* *GG* *AA* *AG* *GG*

6656 (81.03) 1465 (17.84) 93 (1.13) 0.2220 5163 (79.91) 1217 (18.84) 81 (1.25) 0.3277

rs116961268 A/C (D197A) *AA* *AC* *CC* *AA* *AC* *CC*

8141 (99.10) 74 (0.90) 0 (0) 1.0000 6413 (99.23) 50 (0.77) 0 (0) 1.0000

rs185347145 G/A (P548S) *GG* *GA* *AA* *GG* *GA* *AA*

8104 (98.65) 111 (1.35) 0 (0) 1.0000 6374 (98.62) 89 (1.38) 0 (0) 1.0000

____________________________________________________________________________________________________________

Data are numbers of subjects (percentages). H-W *P*, *P* value for Hardy-Weinberg equilibrium.

**Supplementary Table 3.** Association of SNPs with hypertension as determined by multivariable logistic regression analysis.

_____________________________________________________________________________________________________________________________

SNP Dominant Recessive Additive 1 Additive 2

____________________ ____________________ ____________________ ____________________

*P* OR (95% CI) *P* OR (95% CI) *P* OR (95% CI) *P* OR (95% CI)

_____________________________________________________________________________________________________________________________

rs77885682 C/T (R503K) 0.8647 0.4181 0.9721 0.4178

rs2344828 G/A (R697Q) 0.5562 0.1585 0.7878 0.1540

rs2070094 C/T (V488M) 0.0904 0.8829 0.0659 0.5918

rs11669592 A/G 0.1136 0.4007 0.1638 0.2381

rs1601875 T/C 0.0341 1.08 (1.01–1.16) 0.0850 0.0958 0.0355 1.15 (1.01–1.31)

rs3750206 A/T (Y149F) 0.4126 0.2066 0.2574 0.2323

rs17561 C/A (A114S) 0.1906 0.3422 0.1187 0.3831

rs12614237 T/G 0.6108 0.7771 0.6595 0.6661

rs28756992 T/C (S845G) 0.6050 0.6469 0.5669 0.6476

rs149722210 G/A (A1045T) 0.1588 ND 0.1588 ND

rs5035 T/G (D38A) 0.9456 0.3012 0.8148 0.3032

rs150854849 C/T (R179Q) **1.90 × 10^-16^** 6.53 (3.79–12.41) ND **1.90 × 10^-16^** 6.53 (3.79–12.41) ND

rs9651971 G/A 0.2615 0.9969 0.2472 0.9331

rs2867125 G/A 0.0058 0.88 (0.80–0.96) 0.4777 0.0076 0.88 (0.80–0.97) 0.4056

rs80120243 A/G (N224S) 0.1915 0.3285 0.2855 0.2514

rs191775270 G/A (V584M) 0.0954 ND 0.0954 ND

rs257747 C/A 0.6263 0.7495 0.6863 0.6378

rs7134682 G/T 0.0722 0.8320 0.0668 0.3112

rs17107806 G/C (A10P) 0.5840 0.7440 0.5260 0.7726

rs4807160 C/T (E144K) 0.1199 0.0611 0.0236 0.92 (0.85–0.99) 0.2714

rs3735042 T/C (M286T) 0.5279 0.3771 0.4147 0.3929

rs78527125 C/G (L113V) 0.4171 0.3900 0.4727 0.3876

rs62623665 G/A (R352H) 0.2606 ND 0.2606 ND

rs216902 A/G 0.5103 0.9989 0.4946 0.9344

rs143993990 G/A (V1807M) 0.1593 ND 0.1593 ND

rs11877062 T/C (W4R) 0.6315 0.8893 0.6459 0.7202

rs7895803 G/A 0.6357 0.0417 1.11 (1.00–1.23) 0.2274 0.1701

rs3208008 A/C (Q1042H) 0.4823 0.5509 0.5912 0.4473

rs11626651 C/T 0.5725 0.8055 0.6091 0.6374

rs7919322 G/A 0.6542 0.7012 0.5434 0.9393

rs61740658 A/G (E3915G) 0.5864 0.3864 0.4751 0.3968

rs1033423 T/C 0.4525 0.1020 0.3055 0.1056

rs202069030 G/C (R51S) **1.01 × 10^-15^** 0.15 (0.08–0.25) ND **1.01 × 10^-15^** 0.15 (0.08–0.25) ND

rs182546659 T/C (M779V) 0.7123 ND 0.7123 ND

rs117184249 C/T (G2297R) 0.5682 0.1378 0.4773 0.1385

rs7333181 G/A 0.6814 0.5348 0.7335 0.5335

rs34993780 T/G (Y486D) 0.9912 0.5791 0.9002 0.5792

rs117712488 G/T (H889Q) 0.6641 0.2618 0.5736 0.2632

rs6774273 C/T 0.7402 0.8634 0.6824 0.9798

rs6587625 C/T (G197D) 0.5859 0.3763 0.7798 0.3534

rs118014438 C/T (V159I) 0.4987 ND 0.4987 ND

rs3812723 C/T (V396I) 0.0670 0.6629 0.0618 0.6664

rs139320158 C/A (R177M) 0.3791 ND 0.3791 ND

rs10191097 T/G 0.3504 0.6673 0.3995 0.5279

rs10456397 A/T 0.7139 ND 0.7139 ND

rs9834596 A/G 0.0471 1.09 (1.00–1.18) 0.9093 0.0371 1.09 (1.01–1.19) 0.9447

rs2277800 C/T (L28F) 0.1463 0.9688 0.1390 0.8940

rs2769214 A/G 0.9288 0.9159 0.9527 0.9056

rs1952896 T/C 0.4520 0.9371 0.4115 0.8973

rs4581 A/C (L266V) 0.0932 0.1099 0.2023 0.0737

rs9540337 A/C 0.5099 0.1251 0.8152 0.1208

rs78949718 G/A (R269H) 0.7479 ND 0.7479 ND

rs7589293 T/C 0.2232 0.5149 0.2829 0.2815

rs139012426 G/C (S1242T) **7.12 × 10^-11^** 0.24 (0.15–0.38) ND **7.12 × 10^-11^** 0.24 (0.15–0.38) ND

rs2273903 A/G (N148S) 0.6990 0.1322 0.6221 0.1324

rs34273285 G/C (V251L) 0.2539 ND 0.2539 ND

rs138712718 T/G (I7M) 0.8130 0.2252 0.6851 0.2268

rs4246302 A/G 0.6818 0.9176 0.6435 0.9780

rs148087680 C/T (R75Q) 0.0863 ND 0.0863 ND

rs140228894 T/C (L220P) 0.3806 ND 0.3806 ND

rs75432131 G/A (H606Y) 0.0046 1.36 (1.10–1.69) 0.2511 0.0065 1.35 (1.09–1.67) 0.2473

rs12229654 T/G **1.13 × 10^-6^** 0.83 (0.77–0.90) 0.0132 0.82 (0.70–0.96) **1.44 × 10^-5^** 0.84 (0.78–0.91) 0.0014 0.77 (0.65–0.90)

rs1800205 A/G (I134T) 0.9357 0.1799 0.8140 0.1865

rs57075420 C/T (R297C) 0.6443 0.3095 0.7233 0.3088

rs786906 T/C 0.4593 0.7859 0.3779 0.8141

rs116369009 G/A (E152K) 0.8929 ND 0.8929 ND

rs2516399 T/C 0.1664 0.4394 0.1011 0.5571

rs184614603 G/A (R913Q) 0.8812 ND 0.8812 ND

rs11635685 G/C (R275P) 0.4251 0.6691 0.3193 0.9042

rs41301625 T/C 0.3297 0.2923 0.2093 0.3302

rs13019430 G/A 0.9135 0.8740 0.9531 0.8660

rs184127828 A/C (E2796D) 0.8643 0.3564 0.9090 0.3563

rs2290189 C/T 0.4326 0.4293 0.5068 0.4213

rs7305779 A/C (E103A) 0.2330 0.2431 0.3369 0.2256

rs3794153 C/G (N316K) 0.5095 0.9760 0.4913 0.7496

rs139160172 G/A (G139S) 0.7939 0.4800 0.7251 0.4816

rs1808602 A/G 0.4221 0.9825 0.3998 0.7285

rs4896780 A/C 0.3610 0.2189 0.6059 0.1917

rs947211 G/A 0.8630 0.9520 0.8715 0.8883

rs9927623 A/C 0.0256 0.92 (0.85–0.99) 0.7948 0.0228 0.91 (0.84–0.99) 0.2099

rs35737297 T/C (M474V) 0.8790 0.6181 0.8144 0.6207

rs41464348 C/T 0.2409 0.5114 0.3105 0.3449

rs11605924 A/C 0.2492 0.3213 0.3571 0.2704

rs7275212 A/T 0.7113 0.6441 0.6354 0.6571

rs142060916 C/T (S1258L) 0.4308 ND 0.4308 ND

rs150843922 T/C (K703R) 0.5216 ND 0.5216 ND

rs11166240 T/C 0.5459 0.1790 0.8312 0.1741

rs78738447 G/A 0.3991 0.4403 0.3383 0.4437

rs76974938 C/T (D67N) **5.14 × 10^-5^** 0.69 (0.57–0.82) ND **5.14 × 10^-5^** 0.69 (0.57–0.82) ND

rs141576983 G/T 0.9320 0.6462 0.8862 0.6468

rs16914781 A/G 0.9688 0.0753 0.6279 0.0919

rs3869115 G/C 0.0533 0.3543 0.0816 0.2370

rs7157338 G/A 0.2728 0.2879 0.4330 0.1867

rs118147866 C/T (R826W) 0.4662 ND 0.4662 ND

rs201633733 A/G 0.0025 2.80 (1.43–5.73) ND 0.0025 2.80 (1.43–5.73) ND

rs35299879 C/A (E43D) 0.8083 0.4517 0.9038 0.4507

rs26534 G/A 0.2836 0.0545 0.0705 0.4558

rs12352313 A/G (N901S) 0.0086 0.89 (0.81–0.97) 0.0518 0.0240 0.90 (0.82–0.99) 0.0394 0.70 (0.51–0.98)

rs116961268 A/C (D197A) 0.3545 ND 0.3545 ND

rs185347145 G/A (P548S) 0.9962 ND 0.9962 ND

_____________________________________________________________________________________________________________________________

Multivariable logistic regression analysis was performed with adjustment for age and sex. Based on Bonferroni’s correction, *P* values of <1.25 × 10^–4^ (0.05/400) were considered statistically significant and are shown in bold. OR, odds ratio; CI, confidence interval; ND, not determined.

**Supplementary Table 6.** Relation of chromosomal loci, genes, and SNPs identified in the present study to previously examined phenotypes.

| Gene  (chr. locus) | SNP | Nucleotide  (amino acid)  substitution | Previously examined phenotypes |
| --- | --- | --- | --- |
| **Associated with systolic and diastolic BP and hypertension** | | | |
| 12q24.1 | rs12229654 | T/G | Metabolic syndrome (PMID: 25705158), body mass index (PMID: 24861553), glycemic traits (PMID: 23575436), gamma glutamyl transpeptidase (PMID: 21909109), HDL-cholesterol (PMID: 21909109) |
| **Associated with systolic and diastolic BP** | | | |
| *ALDH2* | rs671 | G/A (E504K) | [**Systolic blood pressure**](http://www.ebi.ac.uk/gwas/search?query=Systolic%20blood%20pressure) (PMID: 26390057), [ischemic stroke](http://www.ebi.ac.uk/gwas/search?query=Ischemic%20stroke) (PMID: 26708676), [triglycerides](http://www.ebi.ac.uk/gwas/search?query=Triglycerides) (PMID: 26582766), [primary biliary cirrhosis](http://www.ebi.ac.uk/gwas/search?query=Primary%20biliary%20cirrhosis) (PMID: 26394269), serum alpha-1 antitrypsin levels (PMID: 26174136), body mass index (PMID: 24861553), alcohol dependence, alcohol consumption, and flushing response to alcohol consumption (PMID: 24277619) |
| *ACAD10* | rs11066015 | G/A | Coronary heart disease (PMID: 23364394), [advanced age-related macular degeneration](http://www.ebi.ac.uk/gwas/search?query=Advanced%20age-related%20macular%20degeneration) (PMID: 26691988), [colorectal cancer](http://www.ebi.ac.uk/gwas/search?query=Colorectal%20cancer) (PMID: 26151821), [mean platelet volume](http://www.ebi.ac.uk/gwas/search?query=Mean%20platelet%20volume) (PMID: 22423221), esophageal cancer (PMID: 21642993) |
| *HECTD4* | rs2074356 | C/T | [**Systolic and diastolic blood pressure**](http://www.ebi.ac.uk/gwas/search?query=Systolic%20blood%20pressure) (PMID: 26390057), [thoracic-to-hip circumference ratio](http://www.ebi.ac.uk/gwas/search?query=Thoracic-to-hip%20circumference%20ratio) (PMID: 26675016), [triglycerides](http://www.ebi.ac.uk/gwas/search?query=Triglycerides) (PMID: 26582766), [body mass index](http://www.ebi.ac.uk/gwas/search?query=Body%20mass%20index) (PMID: 25673413), esophageal cancer (PMID: 21642993), gamma glutamyl transpeptidase (PMID: 21909109), HDL-cholesterol (PMID: 21909109), renal function–related traits (PMID: 22797727), glycemic traits (PMID: 23575436) |
| *BRAP* | rs3782886 | A/G | Myocardial infarction (PMID: 24916648), [coronary artery disease or large artery stroke](http://www.ebi.ac.uk/gwas/search?query=Coronary%20artery%20disease%20or%20large%20artery%20stroke) (PMID: 24262325), [primary biliary cirrhosis](http://www.ebi.ac.uk/gwas/search?query=Primary%20biliary%20cirrhosis) (PMID: 26394269), [colorectal cancer](http://www.ebi.ac.uk/gwas/search?query=Colorectal%20cancer) (PMID: 26151821), [body mass index](http://www.ebi.ac.uk/gwas/search?query=Body%20mass%20index) (PMID: 25673413), hematological and biochemical traits (PMID: 20139978) |
| *HECTD4* | rs11066280 | T/A | **Systolic and diastolic blood pressure** (PMID: 26390057), thoracic-to-hip circumference ratio (PMID: 26675016), triglycerides (PMID: 26582766), [body mass index](http://www.ebi.ac.uk/gwas/search?query=Body%20mass%20index) (PMID: 25673413), metabolic syndrome (PMID: 25705158) |
| **Associated with systolic BP** | | | |
| *MUC17* | rs78010183 | A/T (T1305S) | None |
| *OR4F6* | rs141569282 | G/A (A117T) | None |
| *COL6A5* | rs200982668 | G/A (E2501K) | [Body mass index](http://www.ebi.ac.uk/gwas/search?query=Body%20mass%20index) (PMID: 24348519) |
| *MARCH1* | rs61734696 | G/T (Q137K) | [Post bronchodilator FEV1/FVC ratio](http://www.ebi.ac.uk/gwas/search?query=Post%20bronchodilator%20FEV1/FVC%20ratio) (PMID: 26634245), [diisocyanate-induced asthma](http://www.ebi.ac.uk/gwas/search?query=Diisocyanate-induced%20asthma) (PMID: 25918132), [urinary uromodulin levels](http://www.ebi.ac.uk/gwas/search?query=Urinary%20uromodulin%20levels) (PMID: 24578125), [type 2 diabetes](http://www.ebi.ac.uk/gwas/search?query=Type%202%20diabetes) (PMID: 21490949) |
| *PLCB2* | rs200787930 | C/T (E1095K) | [Schizophrenia](http://www.ebi.ac.uk/gwas/search?query=Schizophrenia) (PMID: 25056061, PMID: 21926974) |
| *MOB3C* | rs139537100 | C/T (R24Q) | None |
| *VPS33B* | rs199921354 | C/T (R80Q) | [Type 2 diabetes](http://www.ebi.ac.uk/gwas/search?query=Type%202%20diabetes) (PMID: 24509480) |
| *CXCL8* | rs188378669 | G/T (E31*) | None |
| *COL6A3* | rs146092501 | C/T (E1386K) | [Aging (](http://www.ebi.ac.uk/gwas/search?query=Aging%20(time%20to%20event))PMID: 21782286), [prostate cancer](http://www.ebi.ac.uk/gwas/search?query=Prostate%20cancer) (PMID: 21743467) |
| *ZNF77* | rs146879198 | G/A (R340*) | None |
| *TMOD4* | rs115287176 | G/A (R277W) | None |
| *ADGRL3* | rs192210727 | G/T (R580I) | [Post bronchodilator FEV1/FVC ratio in COPD](http://www.ebi.ac.uk/gwas/search?query=Post%20bronchodilator%20FEV1/FVC%20ratio%20in%20COPD) (PMID: 26634245), [response to antipsychotic treatment in schizophrenia](http://www.ebi.ac.uk/gwas/search?query=Response%20to%20antipsychotic%20treatment%20in%20schizophrenia%20(working%20memory)) (PMID: 21107309), [partial epilepsies](http://www.ebi.ac.uk/gwas/search?query=Partial%20epilepsies) (PMID: 20522523) |
| *PRAMEF12* | rs199576535 | G/A (V341I) | None |
| *PTCH2* | rs147284320 | C/T (V503I) | None |
| *IGSF9B* | rs201459911 | G/A (A1115V) | [Schizophrenia](http://www.ebi.ac.uk/gwas/search?query=Schizophrenia) (PMID: 26198764, PMID: 25056061), [longitudinal alcohol consumption](http://www.ebi.ac.uk/gwas/search?query=Longitudinal%20alcohol%20consumption) (PMID: 26081443), [obesity-related traits](http://www.ebi.ac.uk/gwas/search?query=Obesity-related%20traits) (PMID: 23251661) |
| *RNF213* | rs199976159 | G/A (G222S) | [Lipoprotein-associated phospholipase A_2_ activity change in response to statin therapy](http://www.ebi.ac.uk/gwas/search?query=Lipoprotein-associated%20phospholipase%20A2%20activity%20change%20in%20response%20to%20statin%20therapy) (PMID: 23118302), [moyamoya disease](http://www.ebi.ac.uk/gwas/search?query=Moyamoya%20disease) (PMID: 21048783) |
| 6p21.3 | rs2523638  rs12182351  rs404890 | G/A  T/C  G/T | [Age-related macular degeneration](http://www.ebi.ac.uk/gwas/search?query=Age-related%20macular%20degeneration) (PMID: 22694956), [nevirapine-induced rash](http://www.ebi.ac.uk/gwas/search?query=Nevirapine-induced%20rash) (PMID: 21810746), [chronic lymphocytic leukemia](http://www.ebi.ac.uk/gwas/search?query=Chronic%20lymphocytic%20leukemia) (PMID: 21131588), [nasopharyngeal carcinoma](http://www.ebi.ac.uk/gwas/search?query=Nasopharyngeal%20carcinoma) (PMID: 19664746) |
| *AS3MT* | rs11191454 | A/G | [**Blood pressure**](http://www.ebi.ac.uk/gwas/search?query=Blood%20pressure) (PMID: 24954895), [microalbuminuria](http://www.ebi.ac.uk/gwas/search?query=Microalbuminuria) (PMID: 26631737), [white matter lesion progression](http://www.ebi.ac.uk/gwas/search?query=White%20matter%20lesion%20progression) (PMID: 26451028), [schizophrenia](http://www.ebi.ac.uk/gwas/search?query=Schizophrenia) (PMID: 25056061, PMID: 23974872), [autism spectrum disorder, attention deficit–hyperactivity disorder, bipolar disorder, major depressive disorder, and schizophrenia](http://www.ebi.ac.uk/gwas/search?query=Autism%20spectrum%20disorder,%20attention%20deficit-hyperactivity%20disorder,%20bipolar%20disorder,%20major%20depressive%20disorder,%20and%20schizophrenia%20(combined)) (PMID: 23453885) |
| *CNNM2* | rs12413409  rs11191548 | G/A  T/C | [**Systolic blood pressure**](http://www.ebi.ac.uk/gwas/search?query=Systolic%20blood%20pressure) (PMID: 26390057, PMID: 21909115), [**blood pressure**](http://www.ebi.ac.uk/gwas/search?query=Blood%20pressure) (PMID: 21909110, PMID: 21572416), [coronary artery disease](http://www.ebi.ac.uk/gwas/search?query=Coronary%20artery%20disease) (PMID: 26343387, PMID: 24262325, PMID: 21378988, PMID: 21378990), [large artery stroke](http://www.ebi.ac.uk/gwas/search?query=Coronary%20artery%20disease%20or%20large%20artery%20stroke) (PMID: 24262325), [intracranial aneurysm](http://www.ebi.ac.uk/gwas/search?query=Intracranial%20aneurysm) (PMID: 20364137), [microalbuminuria](http://www.ebi.ac.uk/gwas/search?query=Microalbuminuria) (PMID: 26631737), [white matter lesion progression](http://www.ebi.ac.uk/gwas/search?query=White%20matter%20lesion%20progression) (PMID: 26451028) |
| *NPFFR2* | rs144936999 | G/C (A332P) | [Metabolite levels](http://www.ebi.ac.uk/gwas/search?query=Metabolite%20levels) (PMID: 22916037) |
| *CCHCR1* | rs130075  rs9263739 | C/T (R102Q)  C/T | [Multiple myeloma](http://www.ebi.ac.uk/gwas/search?query=Multiple%20myeloma) (PMID: 23955597), [hematology traits](http://www.ebi.ac.uk/gwas/search?query=Hematology%20traits) (PMID: 23263863), [chronic obstructive pulmonary disease–related biomarkers](http://www.ebi.ac.uk/gwas/search?query=Chronic%20obstructive%20pulmonary%20disease-related%20biomarkers) (PMID: 23144326), [Stevens-Johnson syndrome and toxic epidermal necrolysis](http://www.ebi.ac.uk/gwas/search?query=Stevens-Johnson%20syndrome%20and%20toxic%20epidermal%20necrolysis%20(SJS-TEN)) (PMID: 21912425), [nevirapine-induced rash](http://www.ebi.ac.uk/gwas/search?query=Nevirapine-induced%20rash) (PMID: 21810746), [ulcerative colitis](http://www.ebi.ac.uk/gwas/search?query=Ulcerative%20colitis) (PMID: 19915573) |
| *NT5C2* | rs11191580 | T/C | [**Systolic blood pressure**](http://www.ebi.ac.uk/gwas/search?query=Systolic%20blood%20pressure) **and** [**mean arterial pressure**](http://www.ebi.ac.uk/gwas/search?query=Mean%20arterial%20pressure) (PMID: 26390057), [coronary artery disease](http://www.ebi.ac.uk/gwas/search?query=Coronary%20artery%20disease) (PMID: 26343387), w[hite matter lesion progression](http://www.ebi.ac.uk/gwas/search?query=White%20matter%20lesion%20progression) (PMID: 26451028), [body mass index](http://www.ebi.ac.uk/gwas/search?query=Body%20mass%20index) (PMID: 24861553), [autism spectrum disorder, attention deficit–hyperactivity disorder, bipolar disorder, major depressive disorder, and schizophrenia](http://www.ebi.ac.uk/gwas/search?query=Autism%20spectrum%20disorder,%20attention%20deficit-hyperactivity%20disorder,%20bipolar%20disorder,%20major%20depressive%20disorder,%20and%20schizophrenia%20(combined)) (PMID: 23453885), [schizophrenia](http://www.ebi.ac.uk/gwas/search?query=Schizophrenia) (PMID: 22688191, PMID: 21926974) |
| *HLA-B* | rs1058026 | T/G | [Crohn's disease](http://www.ebi.ac.uk/gwas/search?query=Crohn) (PMID: 26891255), [psoriatic arthritis](http://www.ebi.ac.uk/gwas/search?query=Psoriatic%20arthritis) and [psoriasis vulgaris](http://www.ebi.ac.uk/gwas/search?query=Psoriasis%20vulgaris) (PMID: 26626624), [systemic lupus erythematosus](http://www.ebi.ac.uk/gwas/search?query=Systemic%20lupus%20erythematosus) (PMID: 26606652), [setpoint viral load in HIV-1 infection](http://www.ebi.ac.uk/gwas/search?query=Setpoint%20viral%20load%20in%20HIV-1%20infection) (PMID: 26553974) |
| *C6orf15* | rs2270191  rs2270190 | C/T (V5M)  T/C | [Epstein-Barr virus immune response](http://www.ebi.ac.uk/gwas/search?query=Epstein-Barr%20virus%20immune%20response%20(EBNA-1)) (PMID: 23326239), [hematology traits](http://www.ebi.ac.uk/gwas/search?query=Hematology%20traits) (PMID: 23263863), [Behcet's disease](http://www.ebi.ac.uk/gwas/search?query=Behcet) (PMID: 23001997), [myasthenia gravis](http://www.ebi.ac.uk/gwas/search?query=Myasthenia%20gravis) (PMID: 23055271), [nickel levels](http://www.ebi.ac.uk/gwas/search?query=Nickel%20levels) (PMID: 26025379) |
| *CDSN* | rs117951780 | C/T (S453N) | [Multiple myeloma](http://www.ebi.ac.uk/gwas/search?query=Multiple%20myeloma) (PMID: 23955597), [hematology traits](http://www.ebi.ac.uk/gwas/search?query=Hematology%20traits) (PMID: 23263863), [white blood cell count](http://www.ebi.ac.uk/gwas/search?query=White%20blood%20cell%20count) (PMID: 21738480, PMID: 20139978), [HIV-1 control](http://www.ebi.ac.uk/gwas/search?query=HIV-1%20control) (PMID: 20041166) |
| *CYP17A1* | rs17115100  rs1004467 | G/T  T/C | [**Systolic blood pressure**](http://www.ebi.ac.uk/gwas/search?query=Systolic%20blood%20pressure) (PMID: 26390057, PMID: 19430479), [**mean arterial pressure**](http://www.ebi.ac.uk/gwas/search?query=Mean%20arterial%20pressure) (PMID: 26390057), [coronary artery disease](http://www.ebi.ac.uk/gwas/search?query=Coronary%20artery%20disease) (PMID: 26343387), [microalbuminuria](http://www.ebi.ac.uk/gwas/search?query=Microalbuminuria) (PMID: 26631737), [Parkinson's disease](http://www.ebi.ac.uk/gwas/search?query=Parkinson) (PMID: 19915575) |
| 11p15.5 | rs72655343 | C/A | [Cannabis use](http://www.ebi.ac.uk/gwas/search?query=Cannabis%20use) (PMID: 27023175), [chronic lymphocytic leukemia](http://www.ebi.ac.uk/gwas/search?query=Chronic%20lymphocytic%20leukemia) (PMID: 26956414), [type 2 diabetes](http://www.ebi.ac.uk/gwas/search?query=Type%202%20diabetes) (PMID: 26818947), [glomerular filtration rate in nondiabetics](http://www.ebi.ac.uk/gwas/search?query=Glomerular%20filtration%20rate%20in%20non%20diabetics%20(creatinine)) (PMID: 26831199), [systemic lupus erythematosus](http://www.ebi.ac.uk/gwas/search?query=Systemic%20lupus%20erythematosus) (PMID: 26502338) |
| *PSORS1C1* | rs1063646 | C/T (P133L) | [Parental longevity](http://www.ebi.ac.uk/gwas/search?query=Parental%20longevity%20(mother) (PMID: 27015805), [testicular germ cell tumor](http://www.ebi.ac.uk/gwas/search?query=Testicular%20germ%20cell%20tumor) (PMID: 26503584), [schizophrenia](http://www.ebi.ac.uk/gwas/search?query=Schizophrenia) (PMID: 26198764), [thionamide-induced agranulocytosis in Graves' disease](http://www.ebi.ac.uk/gwas/search?query=Thionamide-induced%20agranulocytosis%20in%20Graves) (PMID: 26151496), [cutaneous lupus erythematosus](http://www.ebi.ac.uk/gwas/search?query=Cutaneous%20lupus%20erythematosus) (PMID: 25827949) |
| *CAT* | rs139421991 | G/A (R320Q) | [Cataracts in type 2 diabetes](http://www.ebi.ac.uk/gwas/search?query=Cataracts%20in%20type%202%20diabetes) (PMID: 23137000) |
| *PSORS1C2* | rs7757012 | T/C | [Multiple myeloma and monoclonal gammopathy](http://www.ebi.ac.uk/gwas/search?query=Multiple%20myeloma%20and%20monoclonal%20gammopathy) (PMID: 26007630), body [height](http://www.ebi.ac.uk/gwas/search?query=Height) (PMID: 25282103), [hematology traits](http://www.ebi.ac.uk/gwas/search?query=Hematology%20traits) (PMID: 23263863), [systemic sclerosis](http://www.ebi.ac.uk/gwas/search?query=Systemic%20sclerosis) (PMID: 21750679) |
| *RNF39* | rs142979264 | C/T | [Chronic lymphocytic leukemia](http://www.ebi.ac.uk/gwas/search?query=Chronic%20lymphocytic%20leukemia) (PMID: 26956414), [anger](http://www.ebi.ac.uk/gwas/search?query=Anger) (PMID: 24489884), [HIV-1 control](http://www.ebi.ac.uk/gwas/search?query=HIV-1%20control) (PMID: 20041166), [AIDS progression](http://www.ebi.ac.uk/gwas/search?query=AIDS%20progression) (PMID: 19115949) |
| *CCDC63* | rs10849915 | T/C | [Drinking behavior](http://www.ebi.ac.uk/gwas/search?query=Drinking%20behavior) (PMID: 23364009, PMID: 21372407), [alcohol consumption](http://www.ebi.ac.uk/gwas/search?query=Alcohol%20consumption) (PMID: 21270382) |
| **Associated with diastolic BP** | | | |
| *ATXN2* | rs7969300 | T/C (N248S) | [Glaucoma](http://www.ebi.ac.uk/gwas/search?query=Glaucoma%20(high%20intraocular%20pressure)) (PMID: 26752265), [systemic lupus erythematosus](http://www.ebi.ac.uk/gwas/search?query=Systemic%20lupus%20erythematosus) (PMID: 26502338) |
| *NAA25* | rs12231744 | C/T (R876K) | [Ischemic stroke](http://www.ebi.ac.uk/gwas/search?query=Ischemic%20stroke) (PMID: 24262325), [hypothyroidism](http://www.ebi.ac.uk/gwas/search?query=Hypothyroidism) (PMID: 22493691), [upper aerodigestive tract cancers](http://www.ebi.ac.uk/gwas/search?query=Upper%20aerodigestive%20tract%20cancers) (PMID: 21437268), [type 1 diabetes](http://www.ebi.ac.uk/gwas/search?query=Type%201%20diabetes) (PMID: 18978792, PMID: 17554300) |
| **Associated with hypertension** | | | |
| *DCLRE1C* | rs150854849 | C/T (R179Q) | [Migraine](http://www.ebi.ac.uk/gwas/search?query=Migraine) (PMID: 23793025) |
| *DUS2* | rs202069030 | G/C (R51S) | None |
| *LOC100505549* | rs139012426 | G/C (S1242T) | [Gamma-glutamyl transferase levels](http://www.ebi.ac.uk/gwas/search?query=Liver%20enzyme%20levels%20(gamma-glutamyl%20transferase)) (PMID: 22001757) |
| *C21orf59* | rs76974938 | C/T (D67N) | None |

Data were obtained from GWAS Catalog (http://www.ebi.ac.uk/gwas). Phenotypes for blood pressure are shown in bold. PMID, PubMed ID; chr., chromosome.
